# Analytical Replication Improves Precision but Does Not Create Environmental Replication: Hierarchical Uncertainty and Decision Stability in Metal(loid) Soil Exposure Screening

**DOI:** 10.3390/toxics14100887

**Published:** 2026-10-02

**Authors:** Antonio Peña-Fernández, Tomás Cámara-Pastor, Gurminderjeet S. Jagdev, Borja Martínez-Alonso, Mark D. Evans, M. Carmen Lobo-Bedmar

**Affiliations:** 1 Area of Legal and Forensic Medicine, Department of Surgery, Medical and Social Sciences, Faculty of Medicine and Health Sciences, University of Alcalá, Ctra. Madrid-Barcelona, Km 33.6, 28871 Madrid, Spain; 2 Leicester School of Allied Health Sciences, De Montfort University, Leicester LE1 9BH, UK; 3 Scientific Computation Research Institute (SCRIUR), University of La Rioja, 26006 Logroño, Spain; 4 Department of Biomedical Sciences, Faculty of Pharmacy, University of Alcalá, Ctra. Madrid-Barcelona, Km 33.6, 28871 Madrid, Spain; 5 Department of Agri-Environmental Research, Institute for Rural, Agricultural and Food Research and Development (IMIDRA), Finca el Encín, Ctra. Madrid-Barcelona, Km 38.2, 28800 Madrid, Spain

**Keywords:** exposure assessment, hierarchical sampling, inferential unit, measurement uncertainty, metal(loid)s, pseudoreplication, soil contamination, toxicological screening

## Abstract

Environmental exposure screening relies on concentration inputs shaped by hierarchical sampling and analytical design. This study investigated how analytical-unit selection and the allocation of replication across a nested soil-sampling hierarchy affect concentration precision, environmental inference, and downstream exposure-screening decisions. We reanalysed a published Leicester/Leicestershire topsoil dataset comprising 26 independent site-level composites, each represented by two physical laboratory samples, with 102 valid analytical determinations overall. Log-scale mixed models quantified variance components for 10 fully detected elements; 500 censor-compatible realisations addressed Cd and Cr; and 10,000 simulations compared reduced analytical designs with the full observed design (R4). Between-composite differences accounted for 65.6–93.8% of log-scale variability, with sensitivity estimates of 87.2% for Cd and 92.3% for Cr. At comparable analytical effort, one determination from each of two physical samples (R3) improved agreement with R4 relative to duplicating one physical sample (R2): median element MdAPE was 1.32% versus 4.27%, mean agreement within ±10% was 96.7% versus 77.5%, and mean rank displacement was 0.47 versus 1.42 positions. Treating the 102 analytical records as independent yielded five FDR-significant urban–rural signals, whereas analyses respecting the 26 independent environmental units yielded none after FDR adjustment. No hazard-index or cancer-risk-band classification switched relative to R4; mean Pb screening-classification switch probabilities were 9.57%, 7.72%, and 0.96% under R1, R2, and R3, respectively. Analytical replication improves measurement precision but does not create environmental replication.

## 1. Introduction

Environmental exposure assessment converts measurements in environmental media into evidence about contact, dose, and potential health concern. This chain begins with a concentration assigned to a location, population or exposure unit, yet that value is not free of uncertainty. Field heterogeneity, composite preparation, physical subsampling, digestion, and instrumental determination can all influence the concentration entering an exposure model. Guidance from the World Health Organization (WHO) and the United States Environmental Protection Agency (US EPA) therefore treats uncertainty and data quality as integral to exposure characterisation rather than as optional qualifications added after risk calculations [1,2].

The measurement result used for an environmental decision is often the endpoint of a hierarchy. A field target may be represented by multiple increments; increments may be pooled into a composite; one or more physical portions may be withdrawn from the composite; and each physical portion may undergo one or more analytical determinations. Contemporary guidance on uncertainty from sampling explicitly includes primary sampling and physical preparation within the measurement process [3,4]. The relative importance of these components is context-dependent. Soil studies have variously found sampling uncertainty to exceed analytical error, laboratory components to dominate under other designs, and both components to change with heterogeneity, particle size, concentration, and sampling protocol [5,6,7]. Consequently, replication is useful only when its location in the hierarchy is stated and aligned with the intended inference. Composite sampling is similarly fit-for-purpose only in relation to its intended estimand: it can provide an area-integrated concentration estimate, but pooling removes increment-level spatial information and therefore cannot quantify within-area field heterogeneity or identify local concentration extremes unless independent field replicates are retained [3,4].

This distinction is also statistical. Independent environmental units support inference about environmental populations or contrasts, whereas repeated preparations or determinations nested within the same environmental unit estimate lower-level variability and can improve its concentration estimate. Treating the latter as if they were additional independent sites is a form of pseudo-replication or inferential-unit error [8]. It can reduce standard errors and *p*-values without adding information about between-site variability. Analytical replication may therefore improve measurement precision while leaving the defensible environmental sample size unchanged [5,6,7,8].

The same hierarchy can propagate into toxicological screening. When exposure and toxicity equations are linear in concentration, modest concentration errors produce proportional changes in continuous hazard or cancer-risk estimates. Decision stability is nevertheless not determined by proportional error alone. A small change close to a threshold can alter a classification, whereas a larger change far from that threshold may have no categorical consequence. The value of additional replication is therefore endpoint-dependent and should be evaluated against both concentration precision and the decisions for which the concentrations are used [1,2,3,4].

Metal(loid)s in composite topsoils provide a useful empirical framework for examining these issues because concentrations are spatially heterogeneous, laboratory preparation involves particulate material, and screening commonly combines several routes and toxicological endpoints. The underlying Leicester and Leicestershire dataset used here has already been characterised for concentrations, spatial patterns, enrichment, and screening-level health implications [9]. The chemical measurements are not presented as newly generated data in the present study. Instead, their nested structure—850 field increments, 26 site-level composites, 52 separately identified physical laboratory samples, and 102 independently processed analytical determinations—allows environmental, physical-subsampling, and analytical components to be examined within one coherent design.

The present study asked how hierarchical analytical-unit selection and replication design affect concentration precision, composite ranking, environmental inference, and downstream exposure-screening decisions. We hypothesised that (i) between-composite variability would dominate the total variance for most elements; (ii) at approximately two determinations per composite, distributing measurements across two physical laboratory samples would be more efficient than duplicating a single physical sample; (iii) treating nested determinations as independent observations would overstate the apparent precision of urban–rural comparisons; and (iv) the effect of reduced replication on screening classification would depend on proximity to the relevant decision threshold.

## 2. Materials and Methods

### 2.1. Previously Published Dataset, Study Area and Purpose of the Present Study

The chemical dataset analysed in this study has already been published and fully characterised [9]. The previous article reported the concentrations, spatial patterns, enrichment characteristics, and screening-level health implications of metal(loid)s in Leicester and Leicestershire topsoils. The present article addresses a different question: whether the level at which replication is allocated and analysed affects concentration precision, environmental inference, and subsequent exposure-screening decisions. No new soil samples were collected, digested, or instrumentally determined for this reanalysis, and the chemical measurements are not presented as newly generated data.

The original dataset was obtained from 18 urban parks or public open spaces in Leicester and eight rural or semi-rural green-space locations in Leicestershire, East Midlands, United Kingdom. The A563 ring road provided the operational boundary used in the original study to distinguish the principal urban domain from the surrounding comparator domain [9]. The rural locations were not assumed to represent pristine geochemical background because soil parent material, drainage, land management, and diffuse anthropogenic inputs could also influence their composition.

Leicester lies within the River Soar valley and is extensively influenced by superficial deposits. Alluvial and river-terrace deposits occur along the principal river corridors, till underlies substantial parts of the urban area, and sporadic glaciofluvial deposits are also present. The wider geological framework includes Triassic Mercia Mudstone Group strata, Lower Jurassic Lias Group units, and Quaternary deposits, while made ground is likely to be extensive in parts of the urban environment [9,10]. These parent materials are relevant particularly to the interpretation of As, V, Fe, Co, and Ni because mudstone-, clay-, and ironstone-associated lithologies can generate naturally elevated or spatially structured concentrations.

The sampled soils also showed heterogeneous physicochemical and textural characteristics. Textures included loam, silty loam, clay loam, and silty clay loam classes, with substantial between-site variation in sand, silt, and clay fractions [9]. All sampled locations were parks, public open spaces or rural/semi-rural green spaces. Urban sites were embedded within the Leicester built environment, whereas rural/semi-rural sites represented comparator green spaces outside the operational urban boundary. Site-specific building density and traffic intensity were not quantified systematically in the historical dataset and were therefore not introduced retrospectively as explanatory covariates. Area type should consequently be interpreted as an operational environmental classification rather than as a quantitative proxy for traffic intensity or degree of urbanisation.

The original field campaign was prospectively designed with predefined sampling effort at both urban and rural locations, resulting in 25 field increments per urban site and 50 per rural/semi-rural site (850 increments overall). For the present reanalysis, however, these increments do not constitute independent environmental observations because they were pooled within each location. The effective environmental sample size is therefore 26 independent site-level composites (18 urban and eight rural/semi-rural). A prospective statistical power/sample-size assessment was undertaken during planning of the original field study, although the original calculation file and detailed assumptions were not retained in the archived project records. For the present secondary analysis, a conventional two-group sensitivity calculation based on the 18 urban and eight rural/semi-rural independent site-level composites indicated that, at α = 0.05 (two-sided) and 80% power, the available design corresponded to a minimum detectable standardized between-group difference of approximately Cohen’s d = 1.24. Accordingly, moderate area-type effects may remain imprecisely estimated, and effect sizes and 95% confidence intervals are reported alongside multiplicity-adjusted tests. The locations were distributed across urban Leicester and surrounding rural/semi-rural Leicestershire to provide broad spatial coverage of contrasting green-space environments within the original regional monitoring framework. The 26 locations did not, however, constitute a probability sample of all soils or land-use categories in Leicester and Leicestershire.

The independent environmental unit throughout the present analysis was the site-level soil composite (hereafter, composite), corresponding to one sampled park or rural area. Field increments, separately identified physical laboratory samples, and independently processed analytical subsamples were nested within each composite. They improved representation or measurement precision but did not constitute additional environmental sampling locations. This hierarchy governed all aggregation, resampling, variance modelling, and inferential analyses.

### 2.2. Original Soil Sampling, Composite Formation, and Sample Preparation

The original field and laboratory procedures are summarised here; their full environmental–geochemistry rationale and application were reported previously [9]. Surface topsoil was collected from May 2017 to March 2018. Twenty-five increments were collected at each of the 18 urban sites along a predefined sampling sequence, with successive points approximately 2 m apart; the first suitable urban point was georeferenced using a handheld Global Positioning System device. Fifty increments were collected using a randomised spatial approach at each of the eight rural/semi-rural sites, and their coordinates were recorded. The different numbers of increments reflected the sampling structure of the two domains: urban parks and public green spaces were generally smaller and more numerous, whereas the rural/semi-rural sampling areas were fewer and substantially larger. Using 50 increments per rural site increased spatial representation within these larger areas while maintaining broadly comparable overall field-sampling effort between the two domains. The resulting dataset comprised 850 increments: 450 urban and 400 rural.

Site-level spatial information was reconstructed from the original field records. Supplementary Table S1 reports the available whole-site park/reserve areas together with the spatial coverage of the sampling design. At each urban site, the prescribed 25-increment cross-shaped design comprised a georeferenced starting point and six additional increments at 2 m intervals along each cardinal direction, giving a nominal horizontal sampling footprint of 288 m^2^ (0.0288 ha) and a maximum extent of 24 m. For the rural/semi-rural sites, spatial coverage was characterised from the 50 recorded field GPS positions at each location.

Each increment was collected from the upper 0–5 cm using a clean plastic shovel, preferentially from exposed soil or ground with sparse vegetation, and was transferred to a labelled, sealed polypropylene bag for transport. Sampling equipment was cleaned between locations. Access to the selected public parks and open spaces was authorised by Leicester City Council. Sampling extended across several seasons and field-weather conditions were not standardised as an experimental factor or incorporated as a covariate. Short-term weather could have influenced surface moisture, runoff, and the spatial distribution of fine surface material, although subsequent air-drying and reporting on a dry-soil basis minimised the direct effect of sample moisture on the reported analytical concentrations. The sampling and pretreatment procedures followed the study-specific protocol described previously [9] and were consistent with the general principles of ISO 18400-102 and ISO 11464 [11,12].

After transport, the soils were spread on clean blotting paper in open plastic trays and air-dried for approximately 2 weeks in a protected laboratory area at ambient room temperature, typically 20–23 °C. Relative humidity varied with ambient laboratory conditions and was not systematically monitored or archived. The dried soils were mechanically disaggregated and passed through a 2 mm stainless-steel sieve to remove stones, coarse fragments, and visible debris; the <2 mm fine-earth fraction was retained for analysis [9,12].

**Figure 1:**
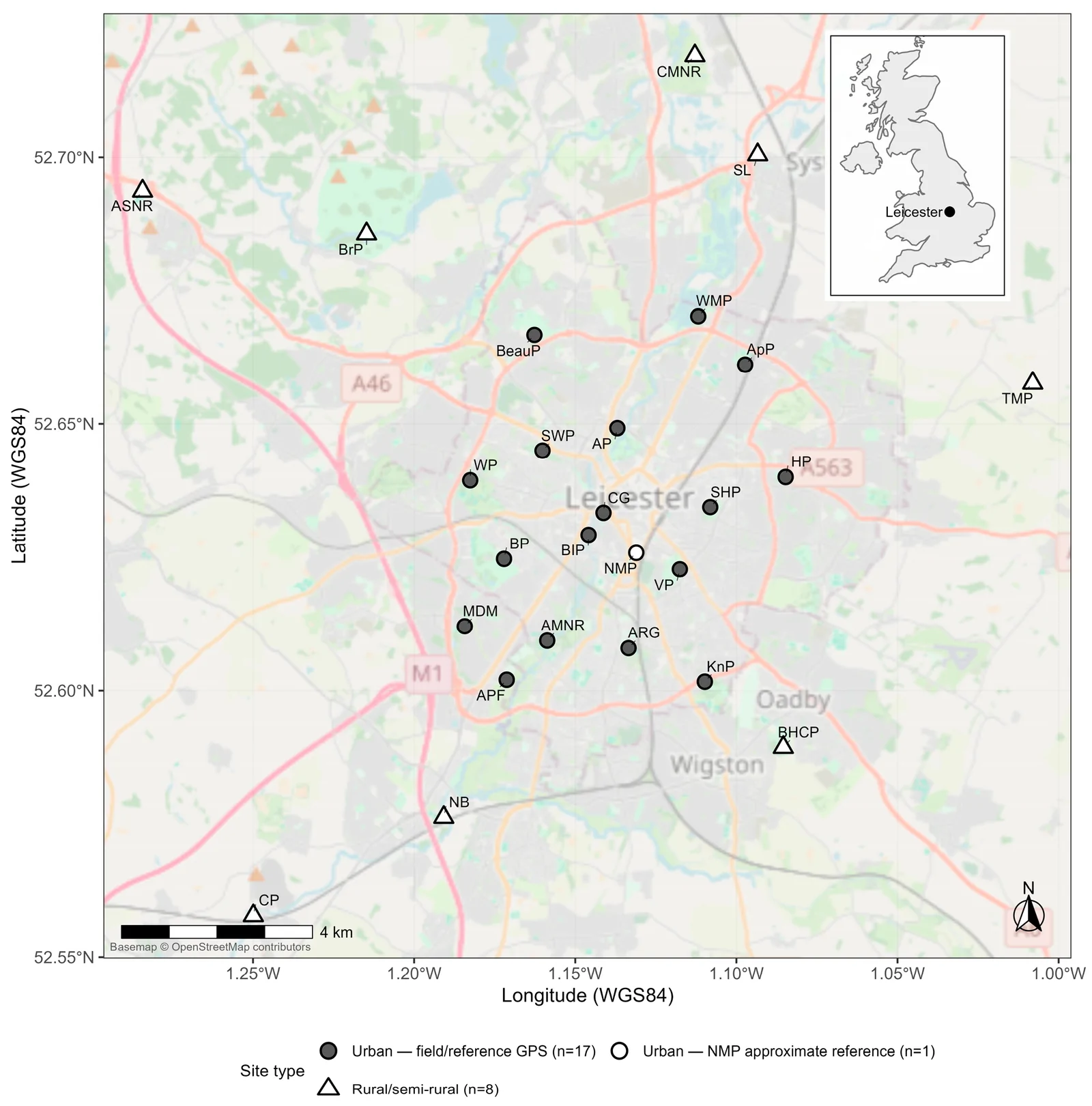
Sampling/reference locations of the 26 independent topsoil composites in Leicester and Leicestershire, United Kingdom. Eighteen urban parks/public open spaces and eight rural/semi-rural green-space locations are shown in WGS84 coordinates. Symbols represent site-level sampling/reference locations and should not be interpreted as the locations of all 850 individual field increments. Nelson Mandela Park (NMP) is shown with a distinct symbol because the original field GPS record could not be recovered and the plotted position represents an approximate park reference location. Site identities, coordinates, area classification, documented park/reserve areas where available, numbers of field increments, and spatial characteristics of the sampling design are provided in Supplementary Table S1. The abbreviations below correspond to the labels shown in the original map artwork; standardized site codes are provided in Supplementary Table S1. Abbreviations: AP, Abbey Park; AMNR, Aylestone Meadows Nature Reserve; APF, Aylestone Playing Fields; ARG, Aylestone Recreation Ground; App, Appleton Park; ASNR, Alterstones Nature Reserve; BeauP, Beaumont Park; BHCP, Brocks Hill Country Park; BIP, Bede Park; BP, Braunstone Park; BrP, Bradgate Park; CG, Castle Gardens; CMNR, Cossington Meadows Nature Reserve; CP, Croft Pasture; HP, Humberstone Park; KnP, Knighton Park; MDM, Mossdale Meadows; NB, Narborough Bog; NMP, Nelson Mandela Park; SHP, Spinney Hill Park; SL, Syston Lakes; SWP, Stokes Wood Park; TMP, The Miles Piece; VP, Victoria Park; WMP, Watermead Park; and WP, Western Park.

Prepared increments from each sampling area were pooled in a pre-cleaned, capped plastic container and intensively homogenised using a motorised rotating mixer. No separate quartering step was used. During progressive transfer of the homogenised material to its storage container, two separately labelled physical laboratory samples were withdrawn: one during the early part of the transfer, and a second toward the end. Material was withdrawn without deliberately targeting a visually distinct portion of the homogenised soil; no formal random-number-based selection procedure was used. Approximate masses of these archived physical samples were not retained in the available records. No separate formal homogeneity test was performed after mixing. Instead, the two physical samples were processed independently, and their between-sample variability provides an empirical measure of residual post-homogenisation heterogeneity. This produced 26 independent site-level composites and 52 physical laboratory samples: 36 urban and 16 rural/semi-rural. By design, each composite represents an area-integrated concentration; pooling therefore precludes direct estimation of increment-to-increment field heterogeneity or local concentration extremes [3,4]. The 26 sampling/reference locations are shown in Figure 1 and listed in Supplementary Table S1.

### 2.3. Microwave Digestion, Instrumental Determination, and Analytical Quality Control

Digestion of the original soil samples was performed at the Institute for Rural, Agricultural and Food Research and Development (IMIDRA), Alcalá de Henares, Madrid, Spain. Approximately 0.500 g of dried, homogenised soil was placed in a closed microwave vessel with 6 mL of 69% HNO_3_ and 2 mL of 37% HCl, both Suprapur^®^ grade (Merck, Darmstadt, Germany). A Multiwave GO microwave reaction system (Anton Paar GmbH, Graz, Austria) was used. The programme comprised a first temperature ramp of 10 °C min^−1^ to 150 °C, a 2 min hold, a second ramp of 3.3 °C min^−1^ to 190 °C, and a final 45 min hold [9,13,14]. After cooling, the digestates were passed through Whatman™ Grade 42 ashless quantitative filter paper (Cytiva, Marlborough, MA, USA) and made up to 50 mL with ultrapure water. The routine ICP-MS working preparation used a 20-fold dilution in 5% *v*/*v* HNO_3_; further dilution was performed when required. Prepared solutions were passed through 0.45 µm polytetrafluoroethylene syringe filters (part 5190-5266; Agilent Technologies, Cheadle, Cheshire, United Kingdom) before instrumental analysis.

Instrumental determinations were undertaken at De Montfort University, Leicester, United Kingdom. Arsenic, Be, Cd, Co, Cr, Cu, Mn, Ni, Pb, V, and Zn were quantified using an Agilent 7900 inductively coupled plasma mass spectrometer with a G3160B integrated autosampler (Agilent Technologies, Santa Clara, CA, USA). The monitored isotopes were ^9^Be, ^51^V, ^52^Cr, ^55^Mn, ^59^Co, ^60^Ni, ^63^Cu, ^66^Zn, ^75^As, ^111^Cd, and ^208^Pb. Prior to sample analysis, instrumental performance was checked using an ICP-MS Tuning Solution (P/N 5185-5959; Agilent Technologies LDA UK Ltd., Cheadle, Cheshire, UK) containing Li, Mg, Y, Ce, Tl, and Co at 1 µg L^−1^. Quantitative calibration solutions were prepared from an Agilent Environmental Calibration Standard (P/N 5183-4688; Agilent Technologies LDA UK Ltd., Cheadle, Cheshire, UK), which contained the ICP-MS target trace elements at 10 mg L^−1^ in 10% HNO_3_. Working standards were prepared at 0, 1, 10, 50, 100, 500, and 1000 µg L^−1^, and calibration curves with coefficients of determination >0.99 were accepted. Calibration standards were included in each analytical run. No separate analyte-specific internal-standard solution added to each sample for quantitative correction is documented in the retained analytical records.

The recorded ICP-MS operating conditions were 1548 W radiofrequency power, an argon plasma-gas flow of 13.9 L min^−1^, a nebuliser-gas flow of 1.0 L min^−1^, an integration time of 0.3 s per point, a dwell time of 10 ms, and three 50 s instrumental acquisitions in pulse-detector mode. Helium collision and ammonia reaction modes were not operational. The repeated instrumental acquisitions were part of a single instrumental measurement and were not counted as independent analytical subsamples.

Iron was the only study element not measured by ICP-MS. It was determined by atomic absorption spectrometry using a PerkinElmer AAnalyst 200 instrument (PerkinElmer, Waltham, MA, USA), using a 0–5 mg L^−1^ calibration range, calibration coefficients of determination of at least 0.999, a routine 100-fold dilution of sample digestates, and a 50-fold dilution of the principal reference-material digest. All concentrations were blank-corrected, converted to the original dry-soil basis, and expressed as mg kg^−1^ dry weight. Because the digestion did not include hydrofluoric acid, results represent the acid-recoverable, or pseudo-total fraction rather than complete dissolution of resistant silicate minerals [9,14].

Quality control covered sample handling, digestion, and instrumental measurement. Sample-contact equipment was detergent-washed, rinsed, acid-cleaned with 5% *v*/*v* HNO_3_, and rinsed repeatedly with ultrapure water; disposable bags, tubes, and filters were used where appropriate. Nine procedural digestion blanks were carried through the complete soil method: one for each of seven soil digestion batches and two with the reference-material runs. For each element, the method limit of detection (LoD) was calculated as three times the standard deviation of the procedural-blank measurements. Results at or below the applicable element-specific LoD, including negative blank-corrected values, were classified as left-censored and were not interpreted as negative environmental concentrations.

Analytical accuracy was evaluated using two soil reference materials processed through the same digestion and measurement workflow. Soil reference materials were selected because they provide a matrix comparable to that of the study topsoils and therefore allow the complete analytical procedure—including digestion recovery, matrix-related effects and instrumental determination—to be evaluated for the 12 target elements, rather than assessing instrumental calibration alone. CRM059 Trace Metals–Loamy Clay 2 (50 g package; product code CRM059-50G), lot LRAA8361 (Sigma-Aldrich/Merck, Darmstadt, Germany), was the principal certified reference material; recoveries across the 12 study elements ranged from 94.9% to 108.6%. NCS DC87101 Soil (National Analysis Center for Iron and Steel/NCS Testing Technology Co., Ltd., Beijing, China) provided a supplementary element-specific check, with recoveries ranging from 33.7% to 108.0%. The assigned Cd value in NCS DC87101 was reference-only, Mn required conversion from MnO, and Fe was assigned as Fe_2_O_3_ (4.69%) and FeO (approximately 0.34%) rather than directly as elemental Fe; an elemental-Fe recovery was therefore not calculated from this material. CRM059 was therefore the principal evidence of analytical accuracy; NCS DC87101 was retained as a transparent secondary check and did not override the matrix-matched CRM059 performance [9]. Element-specific LoDs, detection counts and individual reference-material recoveries are reported in Supplementary Table S2.

The lack of operational collision/reaction modes could not exclude every sample-specific polyatomic interference, particularly for ^52^Cr and, to a lesser extent, ^75^As and ^51^V. This analytical limitation is distinct from the hierarchical-design question examined here. Total Cr was retained in concentration, variance, and ranking analyses, but Cr-specific exposure and toxicological endpoints were not calculated because Cr(III) and Cr(VI) had not been determined separately.

### 2.4. Reconstruction of the Hierarchy, Data Validation, and Multilevel Aggregation

This section and Section 2.5, Section 2.6, Section 2.7, Section 2.8, Section 2.9, Section 2.10, Section 2.11 and Section 2.12 describe the new methodological analysis. Each of the 52 physical laboratory samples was intended to provide two independently processed analytical subsamples. Fifty physical samples had paired determinations, whereas the two physical samples from Brocks Hill Country Park each had one valid determination.

Accordingly, the hierarchy comprised 850 field increments pooled into 26 independent composites, 52 physical laboratory samples—two per composite—and 102 valid analytical determinations. An analytical determination was defined as the concentration result obtained from one independently processed analytical subsample, not as an instrumental reread or one of the repeated ICP-MS acquisitions. The complete hierarchy is summarised in Table 1.

The same composite-level selections were applied simultaneously to all elements within each of 10,000 fixed-seed iterations. R4 is the full analytical-design composite estimate and comparison reference, not the true concentration. The analytical panel comprised As, Be, Cd, Co, Cr, Cu, Fe, Mn, Ni, Pb, V, and Zn; Cd (6/102; 5.88%) and Cr (8/102; 7.84%) were left-censored.

Data restructuring retained, for each valid analytical record, the composite, physical-sample, and analytical-subsample identifiers; area type; element-specific concentration and LoD; and censoring status. Two non-sample rows containing elements and LoD metadata were excluded before reshaping. Structural checks confirmed 26 composites, two physical samples per composite, and 102 analytical records.

The numeric identifier represented a separately labelled physical laboratory sample nested within its composite. The suffixes a and b denoted paired analytical determinations when both were available. The available records did not establish that a and b were chronological first and second analyses. Therefore, a signed b-minus-a contrast was used only as a label-direction quality-control diagnostic and was never described as analytical-order or replicate-order bias.

Three linked concentration representations were constructed:

1. Level A retained all 102 analytical determinations.
2. Level B was obtained by arithmetically averaging the valid determinations within each physical laboratory sample, giving 52 physical sample estimates per element.
3. Level C was obtained by assigning equal weight to the two Level B physical sample means within each composite, giving 26 composite estimates per element.

For composite *i* with physical samples *j* = 1, 2 and *n*_ij_ valid analytical determinations, the Level-C estimate was

*C*_i_ = (1/2) Σ_j=1_^2^ [(1/*n*_ij_) Σ_k=1_^nij^
*y*_ijk_]. (1)

This two-stage estimator was essential for Brocks Hill: it prevented a physical sample with two determinations from receiving twice the weight of a physical sample with only one valid determination.

Cd and Cr required a complete numeric vector for a limited set of deterministic descriptive, aggregation, and paired-agreement operations. For each of these elements, a lognormal distribution was fitted to its positive detected results, and a censored record was assigned the median of that fitted distribution conditional on being below the applicable element-specific LoD. These conditional-median working values were never relabelled as measured observations, were not used as a simple LoD fraction, and were not the primary basis of the Cd/Cr variance analysis. At Levels B and C, a unit was described as censor-affected if at least one contributing determination was censored and as fully censor-affected only if every contributing determination was censored. Detection frequency was reported at Level A only. Element-specific detection information is reported in Supplementary Table S2, and the three analytical levels are retained in the machine-readable supporting data.

### 2.5. Variance-Component Model and Composite-Level Intraclass Correlation

The primary variance decomposition was conducted for the 10 fully detected elements: As, Be, Co, Cu, Fe, Mn, Ni, Pb, V, and Zn. For analytical determination *k* from physical sample *j* nested within composite *i*, the following intercept-only linear mixed model was fitted on the natural-log scale:

ln(*y*_ijk_) = μ + *u*_i_ + *v*_ij_ + ε_ijk_, (2)

where *u*_i_ ~ N(0, σ^2^_composite_) represents variation among the 26 independent composites, *v*_ij_ ~ N(0, σ^2^_physical_) represents variation between the two physical samples within a composite, and ε_ijk_ ~ N(0, σ^2^_analytical_) is the residual difference among independently processed analytical subsamples. The term labelled “analytical” therefore includes remaining subsample-preparation and determination variability below the physical-sample level; it is not limited to instrumental repeatability. Models were fitted by restricted maximum likelihood with lme4 [15].

The contribution of component *h* to total log-scale variance was calculated as

*P*_h_ (%) = 100σ^2^_h_/(σ^2^_composite_ + σ^2^_physical_ + σ^2^_analytical_). (3)

The composite-level intraclass correlation coefficient was

ICC_composite_ = σ^2^_composite_/(σ^2^_composite_ + σ^2^_physical_ + σ^2^_analytical_). (4)

Thus, ICC_composite_ estimates the proportion of total log-scale variation attributable to genuine differences among the independent composites rather than to variation between physical samples or among analytical subsamples [16].

Uncertainty about the fully detected elements was assessed using 1000 nonparametric cluster-bootstrap resamples. Entire composites were sampled with replacement, and every physical sample and analytical determination belonging to a selected composite was retained. Analytical rows were never resampled as though independent. This preserves within-composite dependence and treats the composite as the resampling unit [17]. The model was refitted to every resample; fit status and singularity were recorded. Percentile 95% confidence intervals were defined by the 2.5th and 97.5th percentiles of successful fits. The corresponding variance estimates are presented in Section 3.2.

### 2.6. Censor-Compatible Variance Sensitivity Analysis for Cd and Cr

Cd and Cr were the only left-censored elements: 6/102 Cd results (5.88%) and 8/102 Cr results (7.84%) were at or below their applicable LoDs. The raw result, applicable element-specific LoD, and censoring indicator were retained for every record. Fixed replacement by LoD/2 or LoD/√2 was not used as the principal procedure because single-value substitution can distort environmental distributions, associations and variance components [18,19].

For each element, μ̂ and σ̂ were estimated from the natural logarithms of its positive detected concentrations. Five hundred complete, censor-compatible realisations were generated. If *L*_i_ was the LoD of censored record *i* and *F*LN was the cumulative distribution function of the fitted lognormal distribution, the draw was generated as

*U*_i_^(m)^ ~ Uniform [0, *F*LN(*L*_i_; μ̂, σ̂)],

*Y*_i_^(m)^ = *F*LN^−1^[*U*_i_^(m)^; μ̂, σ̂], (5)

so every imputed realisation was positive and no greater than the applicable LoD. Detected results were held at their measured values. The model in Equation (2) was fitted independently to each completed dataset.

Across successful fits, each variance component, its percentage contribution, and ICC_composite_ were summarised by the median and 2.5th–97.5th percentile range. These percentiles quantify sensitivity to plausible concentrations below the LoD; they are not sampling confidence intervals and must not be interpreted as the composite-cluster bootstrap intervals used for fully detected elements. A minimum of 95% successful model fits was prespecified, and variability across realisations was confirmed.

### 2.7. Reproducibility at the Analytical-Subsample and Physical-Sample Levels

Agreement was evaluated separately at two levels of the hierarchy. Analytical-subsample agreement used the 50 physical samples with paired a/b determinations. Physical-sample agreement was evaluated after averaging valid determinations within each physical sample and then comparing physical sample 1 with physical sample 2 in all 26 composites. For Cd and Cr, these descriptive paired calculations used the conditional-median working values defined in Section 2.4.

For a pair *x* and *y*, relative percent difference was calculated as recommended for duplicate-based measurement comparisons [3,4]:

RPD (%) = 100|*x* − *y*|/[(*x* + *y*)/2]. (6)

For each element and hierarchy level, the following were reported: number of valid pairs; median, interquartile range, 95th percentile, and maximum RPD; median absolute difference; median log ratio; median multiplicative difference; Spearman rank correlation; and Lin concordance correlation coefficient (CCC) on both the original and log scales [20]. Log-scale CCC was designated the principal concordance statistic because the variance decomposition and Bland–Altman analyses used log concentrations. Bland–Altman plots for the analytical pairs displayed log pair difference against log pair mean for all 12 elements [21].

Paired Wilcoxon signed-rank tests were used to evaluate systematic direction within pairs, with Benjamini–Hochberg false-discovery-rate (FDR) adjustment across the element family [22]. For analytical pairs, the direction was b versus a and was interpreted only as label direction because chronological order was unknown. For physical-sample pairs, the direction corresponded to the recorded physical-sample labels and did not imply a preparation sequence.

Pre-analysis checks identified four highly discordant analytical element-pairs: Ni and Cr in physical sample 3760 from Syston Lakes (SL), Zn in sample 3759 from Syston Lakes (SL), and Zn in sample 3819 from Braunstone Park (BP). They were retained in every primary analysis. Exact leave-one-extreme-pair-out sensitivity analyses quantified their effect on the relevant variance component and on the comparison of R2 with R3. When removal of both determinations made a two-physical-sample estimate undefined for one composite–element combination, that combination was omitted symmetrically from R2, R3, and R4 for that sensitivity analysis only. Agreement results are reported in Supplementary Table S2 and Figures S1 and S2; the prespecified extreme-pair sensitivity analyses are reported in Supplementary Table S3.

### 2.8. Monte Carlo Evaluation of Alternative Replication Designs

The reduced-design analysis used repeated resampling from the observed hierarchy rather than generating a new parametric population of detected concentrations. Its aim was to compare how the same observed analytical effort would perform when placed at different levels of this dataset. Following the principle that simulations should define their aim, data-generating mechanism, estimand, methods, and performance measures explicitly [23], four designs were evaluated over 10,000 iterations using a fixed pseudorandom seed:

**R1.**  *Minimal design: one of the two physical samples was selected at random within each composite, and one valid analytical determination was selected from that physical sample.*

**R2.**  *Analytical replication prioritised: one physical sample was selected at random, and all of its valid analytical determinations were averaged. It normally used two determinations; because each Brocks Hill physical sample had only one, the observed expected effort was 1.962 determinations per composite.*

**R3.**  *Physical-sample coverage prioritised: one valid analytical determination was selected independently from each of the two physical samples, and the two results were averaged. This required exactly two determinations per composite.*

**R4.**  *Full analytical design: all valid determinations were averaged within each physical sample, and the two resulting physical-sample means were then averaged with equal weight. Its observed effort was 102/26 = 3.923 determinations per composite.*

R2 and R3 were the prespecified central comparison because they required almost identical analytical effort but allocated it differently: repeated determinations within one physical sample in R2 versus one determination from each of two distinct physical samples in R3. Selections for R1 and R2 were performed independently rather than forcing the two designs to use the same selected physical sample.

Within each composite, design, and iteration, the selected physical sample or analytical record was applied jointly to all 12 elements. This preserved the observed multielement structure and ensured that a simulated concentration vector corresponded to one coherent record selection rather than to element-by-element mixing. Detected values were resampled only through their observed hierarchical records. For Cd and Cr, new censor-compatible draws below the applicable element-specific LoD were generated in every iteration using Equation (5) before R1–R4 were calculated; censoring uncertainty was thereby propagated through both the reduced designs and the full design.

R4 was used as the full analytical-design composite estimate, not as a true, certified, or error-free composite concentration. For the 10 fully detected elements, R4 was invariant across iterations; for Cd and Cr, it varied only because new plausible values below the LoD were drawn in each iteration. Every comparison between R1–R3 and R4 was matched by composite and iteration.

For element *e*, composite *i*, reduced design *d*, and iteration *m*, relative error was

*r*_ei_d_m_ = (*C*_ei_d_m_ − *C*_ei_R4_m_)/*C*_ei_R4_m_. (7)

The principal concentration-performance measures, pooled over all composite–iteration cells for each element and design, were

RMSE_e_d = √{mean[(*C*_ei_d_m_ − *C*_ei_R4_m_)^2^]}, (8)

relative RMSE_e_d (%) = 100√[mean(*r*_ei_d_m_^2^)], (9)

MdAPE_e_d (%) = 100 median(|*r*_ei_d_m_|), (10)

and the proportions satisfying |*r*_ei_d_m_| ≤ 0.05, 0.10, and 0.20. Absolute RMSE was retained in the original element-specific concentration units, whereas the relative measures supported comparison across elements with different concentration scales. Equation (10) defines the median absolute percentage error (MdAPE), which is distinguished throughout from mean absolute percentage error [23].

R2 and R3 were also evaluated in the prespecified leave-one-extreme-pair-out analyses described in Section 2.7. Because a single large concentration can dominate squared error, RMSE, MdAPE, and threshold agreement were considered jointly. Accordingly, no design was defined as universally superior on the basis of one metric alone.

Element-specific R2–R3 performance and extreme-pair sensitivity results are provided in Supplementary Table S3. Complete simulation outputs are supplied as machine-readable supporting data.

### 2.9. Stability of Composite Ranking and Sensitivity to the Chosen Inferential Unit

Within every element, design, and iteration, the 26 composites were ranked from highest to lowest concentration, with average ranks assigned to ties. Spearman agreement with R4 was calculated as the Pearson correlation between the corresponding rank vectors; Kendall’s *tau* and absolute composite-rank displacement were also obtained. Mean absolute rank displacement was

MRD_e_d = mean(|Rank_ei_d_m_ − Rank_ei_R4_m_|). (11)

Element-specific rank summaries are reported in Supplementary Table S3 and Figure S3.

Inferential-unit choice was examined directly using the observed full dataset. Three deliberately contrasting analyses were performed: (i) a naïve Mann–Whitney comparison treating the 102 analytical records as independent observations; (ii) a Mann–Whitney comparison using the 26 independent full-design composite estimates; and (iii), for the 10 fully detected elements, a cluster-aware model. Cliff’s delta for the two rank-based comparisons was derived from the Mann–Whitney statistic *U* as

δ = 2*U*/(*n*urban × *n*rural) − 1, (12)

with positive values indicating generally higher urban ranks [24]. The cluster-aware model was

ln(concentration) ~ area type + (1|composite) + (1|composite:physical sample).(13)

The mixed models were fitted by maximum likelihood, and the model containing area type was compared with its intercept-only counterpart by a likelihood-ratio test. The exponentiated fixed-effect coefficient was interpreted as the estimated urban-to-rural concentration ratio. FDR adjustment was applied across elements separately within each inferential approach. The 102-record analysis was included only to demonstrate the consequence of treating analytical replication as environmental replication; it was not regarded as a valid environmental comparison. Results are reported in Supplementary Table S4.

### 2.10. Previously Defined Exposure and Toxicological Screening Framework

The exposure model was kept identical to that used in the published Leicester/Leicestershire topsoil assessment [9]. Receptor definitions, exposure routes, toxicity values, conversion factors, and classification thresholds were unchanged. Only the composite-specific concentration vector was replaced by its R1, R2, R3, or R4 representation. Before propagation, all constants were verified against the files supporting the previous assessment.

Three receptor scenarios were retained. The child resident/public-access scenario used a soil ingestion rate (IRS) of 200 mg day^−1^, body weight (BW) of 15 kg, exposure frequency (EF) of 365 days year^−1^, exposure duration (ED) of 6 years, exposure time (ET) of 24 h day^−1^, exposed skin area (SA) of 2800 cm^2^, and soil adherence factor (AF) of 0.2 mg cm^−2^. The adult resident/public-access values were 50 mg day^−1^, 70 kg, 365 days year^−1^, 30 years, 24 h day^−1^, 5700 cm^2^, and 0.2 mg cm^−2^, respectively. The supplementary adult outdoor/maintenance-worker scenario used 50 mg day^−1^, 70 kg, 250 days year^−1^, 25 years, 8 h day^−1^, 3300 cm^2^, and 0.2 mg cm^−2^, respectively, and was evaluated only for the 18 urban sites.

Non-cancer averaging time equalled exposure duration: 2190, 10,950, and 9125 days, or 52,560, 262,800, and 219,000 h, for the child, adult resident, and worker, respectively. Cancer averaging time was 70 years: 25,550 days, or 613,200 h. All exposure and toxicity inputs are listed in Supplementary Table S5.

For soil concentration *C*s (mg kg^−1^), route-specific non-cancer doses were calculated using the previously reported screening equations [25,26]:

ADD_oral_ = (*C*s × IRS × EF × ED × RBA × 10^−6^)/(BW × AT_nc,days_), (14)

ADD_dermal_ = (*C*s × SA × AF × ABS_d_ × EF × ED × 10^−6^)/(BW × AT_nc,days_), (15)

where RBA was relative oral bioavailability, fixed at 1, and ABS_d_ was the element-specific dermal absorption fraction where available. The soil-particle contribution to air was represented as

*C*_air_ = *C*s × PM_10_ × *f*_resp_ × 10^−6^, (16)

where PM_10_ was fixed at 19.5025 µg m^−3^ and the respirable fraction *f*_resp_ at 0.5. *C*_air_ is expressed as µg analyte m^−3^. The time-weighted inhalation concentration was

*C*_inh,nc_ = (*C*_air_ × ET × EF × ED)/AT_nc,hours_. (17)

This preserves the PM_10_-based structure of the previous study and does not substitute a generic soil particulate-emission factor.

Oral and dermal hazard quotients were obtained by dividing ADD by the applicable oral reference dose (RfD_o_). The inhalation hazard quotient was calculated by dividing the time-weighted concentration in µg m^−3^ by 1000 times the inhalation reference concentration expressed in mg m^−3^:

HQ_oral_ = ADD_oral_/RfD_o_

HQ_dermal_ = ADD_dermal_/RfD_o_;

HQ_inh_ = *C*_inh,nc_/(1000 × RfC_i_). (18)

The hazard index (HI) was the sum of all included element- and route-specific HQs. For carcinogenic screening, oral and dermal lifetime average daily doses used AT_ca,days_ in place of AT_nc,days_ and were multiplied by the relevant oral slope factor. The inhalation contribution was

CR_inh_ = [(*C*_air_ × ET × EF × ED)/AT_ca,hours_] × IUR, (19)

where IUR was the inhalation unit risk per µg m^−3^. Total cancer risk (CR) was the sum of the applicable oral, dermal, and inhalation components [25,26].

The same element-specific decisions as in the published study were retained. Total As was conservatively treated as inorganic As for screening, without claiming that all soil As was inorganic or bioaccessible. Cr remained in measurement-design analyses but was excluded from Cr-specific HQ, HI, and CR calculations because oxidation-state speciation was unavailable. Fe had no toxicity endpoint in the predefined model. Total V contributed to non-cancer assessment but was not assigned the carcinogenic potency of V_2_O_5_. Pb was excluded from a conventional RfD-based HQ and from the summarised CR framework. It was evaluated separately as *C*s/200 mg kg^−1^ for child and adult resident/public-access scenarios, consistent with the 2025 US EPA Residential Lead Directive, and as *C*s/800 mg kg^−1^ for the worker scenario [26,27]. These Pb quantities are concentration screening ratios, not HQs, blood-lead predictions, UK statutory park criteria, or site-specific risk estimates.

### 2.11. Propagation of Replication-Design Uncertainty into Screening Outcomes

In every iteration, the complete 12-element concentration vector generated for each composite under R1–R4 was passed through the previously defined model in Section 2.10. No receptor or toxicity parameter was resampled. This design isolated the consequence of concentration representation and replication allocation from broader parameter, behavioural, toxicokinetic, or population variability.

For each composite, receptor, design, and iteration, the workflow retained element- and route-specific HQs; route-specific and total HIs; route-specific and total CR; the element contributing most strongly to total HI; the element contributing most strongly to total CR; and the separate Pb screening ratio. Composite-level medians and 2.5th–97.5th percentiles across iterations therefore describe measurement-design sensitivity. They are not probabilistic estimates of population exposure or health risk. 

Classification stability was calculated by comparing each R1–R3 result with its composite- and iteration-matched R4 result. HI was classified as <1 or ≥1. CR was divided into <10^−6^, 10^−6^–10^−4^ inclusive, and >10^−4^. Pb was classified as a screening ratio < 1 or ≥1. For each reduced design, the workflow calculated the composite-specific probability of changing category relative to R4, together with the mean and maximum switch probability across eligible composites. It also calculated whether the principal HI or CR driver remained the same. Because the child and adult resident/public-access scenarios used the same 200 mg kg^−1^ Pb comparator, their Pb switch probabilities were identical. Worker results were limited to the 18 urban sites.

Adult-worker results and principal-driver stability are reported in Supplementary Table S6.

All outcomes are interpreted as screening-level sensitivity to analytical design. They do not constitute site-specific risk assessments, estimates of absorbed dose, evidence of clinical risk, or confirmation that an individual site is safe or unsafe.

### 2.12. Statistical Software, Multiplicity, and Reproducibility

All analyses were performed in R version 4.4.2 [28], principally using the packages *lme4*, *dplyr*, *tidyr*, *ggplot2*, and *readxl*. Statistical tests were two-sided, with *p* < 0.05 considered statistically significant. The Benjamini–Hochberg procedure was applied within each defined family of element-wise comparisons to control the false discovery rate [22]. Cluster-bootstrap confidence intervals, censoring-sensitivity percentile ranges, and Monte Carlo percentile ranges are reported separately because they quantify different sources of uncertainty.

All stochastic procedures used fixed and reproducible seeds. The reduced-design analysis comprised 10,000 iterations, the complete-composite cluster bootstrap comprised 1000 resamples, and the Cd and Cr censoring-sensitivity analysis comprised 500 realisations. Before analysis, the hierarchical structure, censoring indicators, element-specific limits of detection, and exposure-model constants were verified against the original records. Mixed-model fits were evaluated for convergence and singularity. The hierarchical data and R code required to reproduce the reported analyses are provided in the Supplementary Materials.

## 3. Results

### 3.1. Hierarchical Dataset and Analytical Reproducibility

All 850 field increments were represented in 26 independent composites: 18 urban and eight rural. The composites yielded 52 physical laboratory samples and 102 analytical determinations. Fifty physical samples had paired determinations and two had one determination, producing average analytical efforts of 1.962 determinations per composite under R2 and 3.923 under R4 (Table 1).

Median analytical-duplicate RPDs were low, ranging from 0.60% for Fe to 5.33% for Cd, and log-scale CCCs ranged from 0.884 for Zn to 0.997 for Pb (Table 2; Supplementary Table S2). Physical-sample median RPDs were consistently larger, from 5.97% for Fe to 10.56% for Cu, while log-scale CCCs ranged from 0.655 for Mn to 0.941 for Cu (Supplementary Table S2). The a-versus-b label-direction tests were FDR-significant for all 12 elements. Median b-versus-a differences were negative for 11 elements (−5.12% to −1.54%) and +0.22% for Fe. This pattern is a label-direction QA signal only because a/b chronology was not documented. At the physical-sample level, only Pb retained a sample-direction offset after FDR adjustment (*q* = 0.015).

The main analysis retained the four prespecified extreme pairs from Syston Lakes and Braunstone Park. Their high pairwise RPDs chiefly affected analytical residual variance for the relevant element rather than overturning the dominant between-composite component (Supplementary Table S3).

### 3.2. Partitioning Environmental, Physical-Subsampling and Analytical Variability

**Figure 2:**
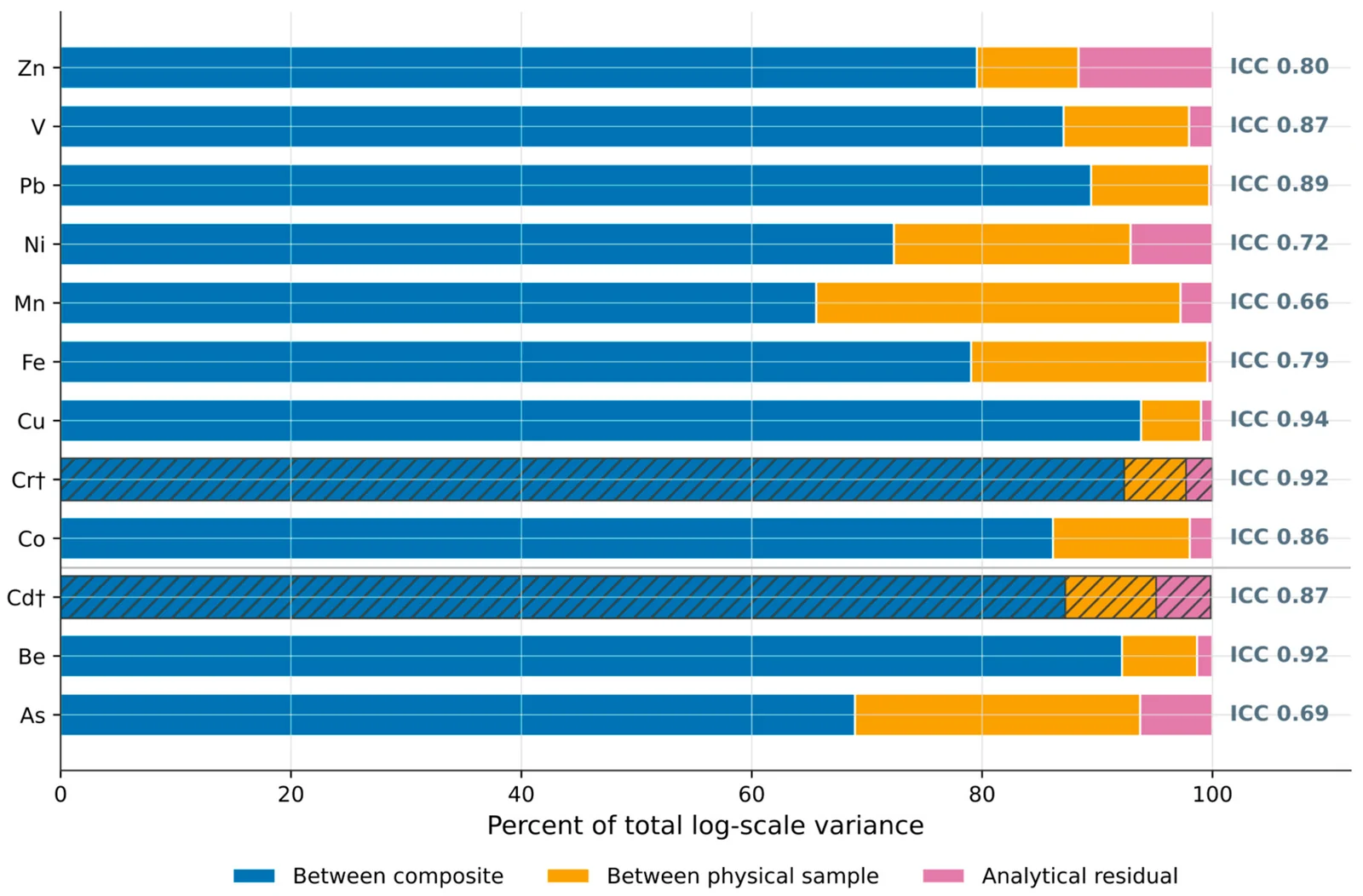
Partitioning of total log-scale variance among between-composite, between-physical-sample, and analytical-residual components for 12 elements. For the 10 fully detected elements, components were estimated using nested log-scale linear mixed models. Cd and Cr (†) show median estimates across 500 censor-compatible realisations. Diagonal black hatching identifies the Cd and Cr bars to distinguish these censoring-sensitive estimates from those of the fully detected elements. ICC denotes the composite-level intraclass correlation coefficient. Component percentages may not sum to exactly 100% because of rounding and, for Cd and Cr, separate summarisation across realisations.

Between-composite differences dominated log-scale variability for every fully detected element, accounting for 65.6% (Mn) to 93.8% (Cu) of total variance (Table 2; Figure 2). The corresponding composite ICCs were 0.656 for Mn, 0.690 for As, 0.723 for Ni, 0.790 for Fe, 0.795 for Zn, 0.861 for Co, 0.871 for V, 0.895 for Pb, 0.921 for Be and 0.938 for Cu. Between-physical-sample variability contributed 5.2–31.6%, whereas analytical residual variability contributed 0.28–11.6%. The widest bootstrap ICC intervals occurred for Mn and As, consistent with the limited number of independent composites and their larger lower-level variance components.

The 500-realisation sensitivity analysis gave median between-composite proportions of 87.2% for Cd and 92.3% for Cr, with ICCs of 0.872 (realisation interval 0.830–0.896) and 0.923 (0.906–0.931), respectively (Figure S4). Thus, the conclusion that composite-to-composite heterogeneity was not dependent on fixed substitution of censored values. All 500 models fit for each censored element, and variability across realisations was confirmed.

### 3.3. Efficiency of Alternative Replication Designs

**Figure 3:**
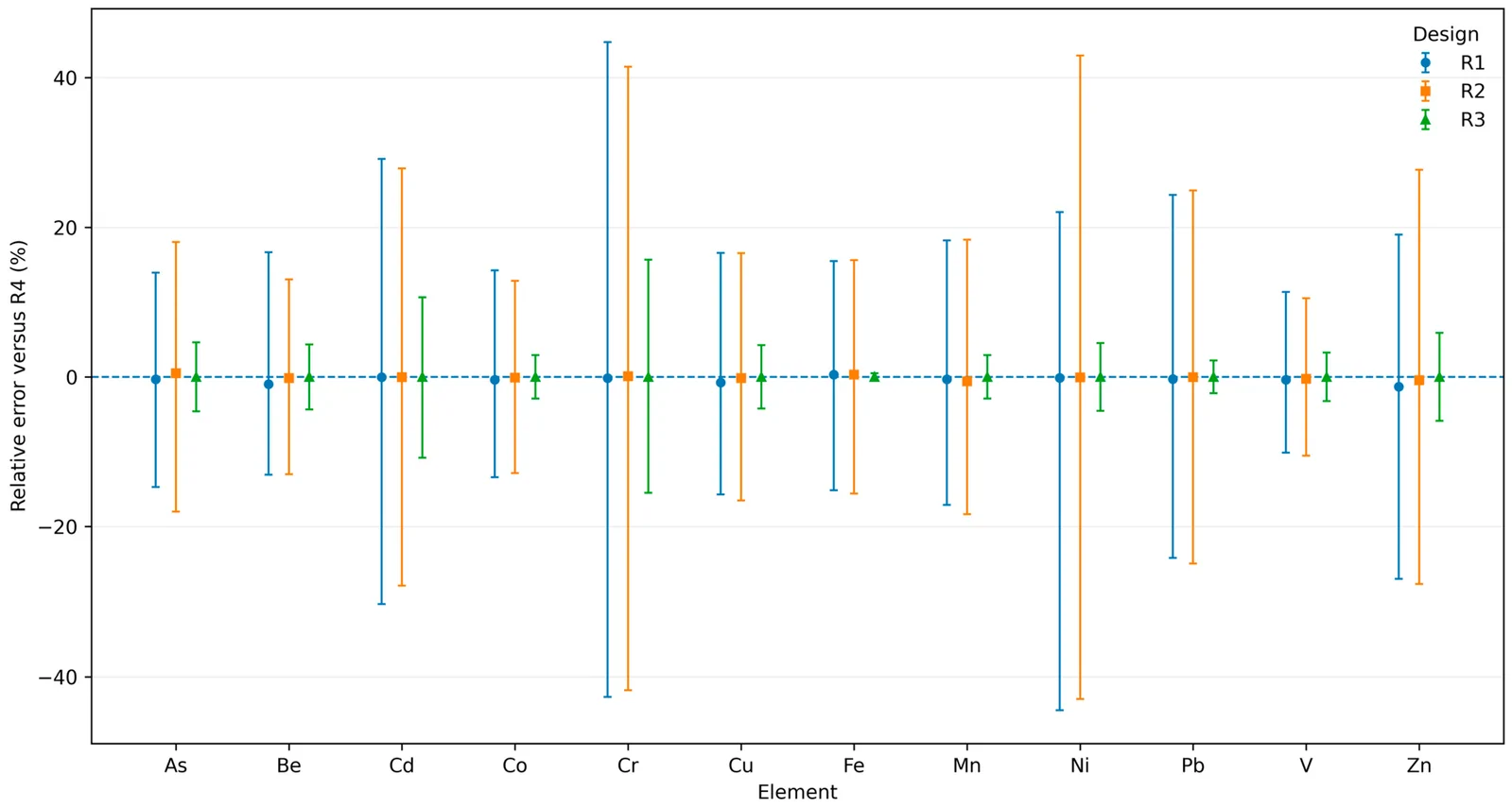
Concentration uncertainty under reduced analytical designs relative to the full observed design (R4). Points show the median relative error and vertical bars the 5th–95th percentile interval across matched composite-iteration cells for R1, R2, and R3. R2 averages all valid determinations from one selected physical sample, whereas R3 averages one determination from each of the two physical samples. The dashed line indicates zero relative error.

At essentially equivalent effort, R3 generally performed better than R2 on the prespecified concentration-error and rank-stability summaries (Table 3; Figure 3). Across the 12 elements, the median of element-specific MdAPEs was 4.27% for R2 and 1.32% for R3. Mean probability of agreement within ±10% of R4 increased from 77.5% to 96.7%, mean Spearman correlation increased from 0.953 to 0.991, and mean absolute rank displacement decreased from 1.42 to 0.47 positions (Supplementary Table S3; Figure S3). R1 was least efficient overall, with median MdAPE 4.91%, mean ±10% agreement 74.3%, mean Spearman correlation 0.948, and mean rank displacement 1.50 positions.

For MdAPE and ±10% agreement, R3 performed better than R2 for every element. The magnitude varied, however: R3 MdAPE ranged from 0.21% for Fe to 2.33% for Cd. Leave-one-extreme-pair-out analyses supported the general R3 advantage for MdAPE and ±10% agreement, but not an absolute claim across every metric. After exclusion of either extreme Zn pair, R3 relative RMSE was approximately equal to or slightly greater than R2, whereas MdAPE and ±10% agreement still favoured R3. The result therefore describes an allocation tendency under this hierarchy, not universal dominance of R3 for all error criteria and data structures.

### 3.4. Analytical Unit and Environmental Inference

Inferential conclusions changed when the number of analytical records was confused with the number of independent composites. The deliberately naïve *N* = 102 analysis produced FDR-significant urban–rural differences for As, Cr, Cu, V, and Zn. When the same concentration information was represented by the 26 independent R4 composite estimates, no element remained significant after FDR adjustment. For V, which had the smallest composite-level *q* value, Cliff’s *δ* was −0.528 (95% CI: −0.917 to −0.042; raw *p* = 0.0348; *q* = 0.417). The cluster-aware model likewise estimated an urban:rural concentration ratio of 0.759 (95% CI: 0.607–0.950; raw *p* = 0.0181; *q* = 0.181). These results illustrate that loss of FDR significance does not imply absence of an area-type effect; rather, the effect estimates remain subject to substantial uncertainty with 18 urban and eight rural sites (Supplementary Table S4; Figure S5).

The directions and descriptive medians were broadly similar across representations—for example, higher urban Cr, Cu, and Zn and higher rural As and V—but the evidential precision was not. The FDR significance of the five naïve signals was contingent on counting nested analytical information as if it supplied 102 independent environmental units; none remained FDR-significant when nesting was respected. Analytical replication increased apparent precision; it did not create environmental replication.

### 3.5. Propagation into Exposure and Toxicological Screening

**Figure 4:**
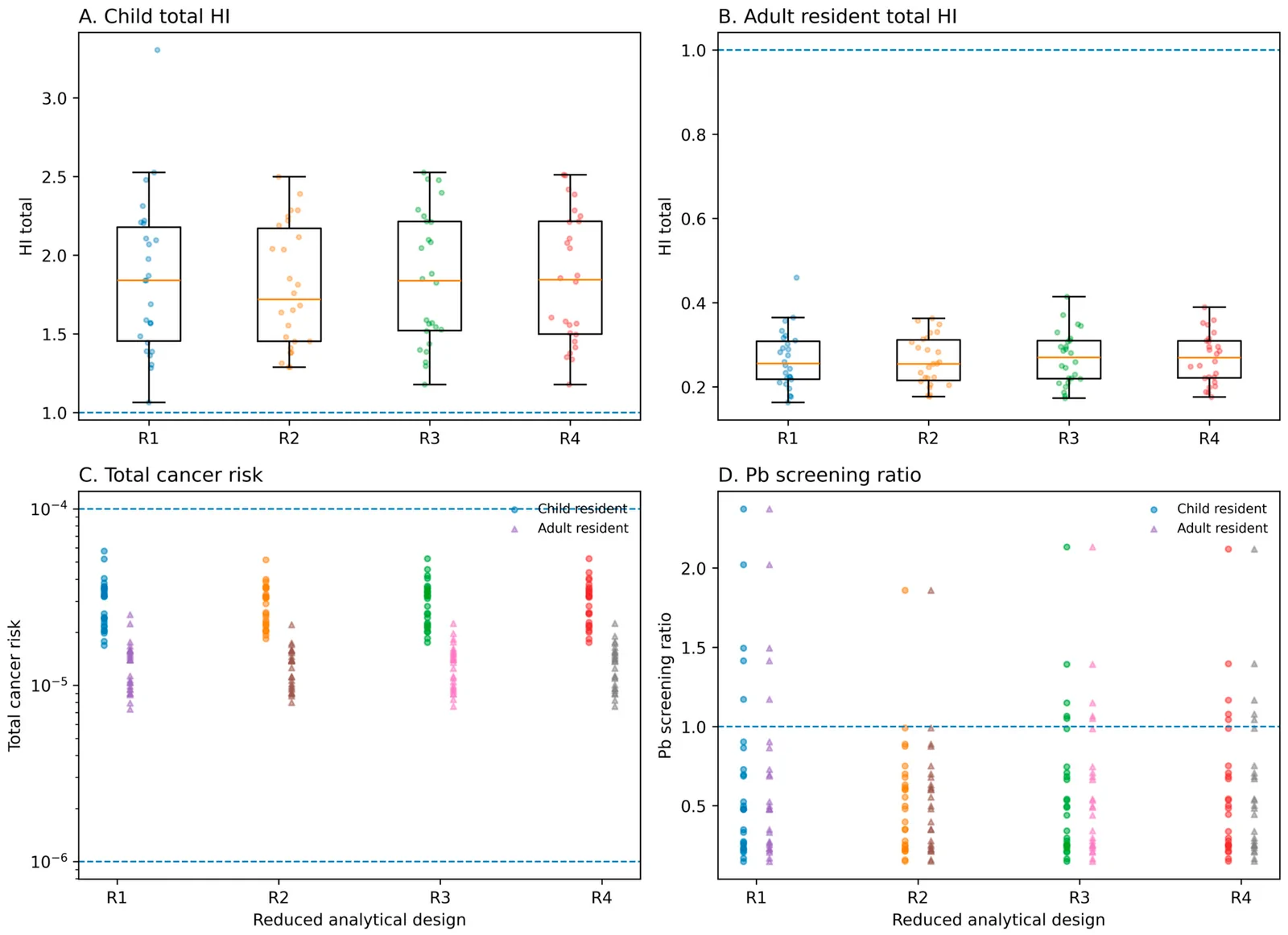
Propagation of analytical-design uncertainty into resident/public-access exposure-screening endpoints. Panels show composite-specific medians across 10,000 iterations for child hazard index HI (**A**), adult HI (**B**), total cancer risk CR (**C**), and the Pb screening ratio (**D**) under R1-R4. Colours distinguish the four analytical designs (R1–R4) consistently across panels; in panels C and D, symbol shape additionally distinguishes child- and adult-resident scenarios. Points represent composites; boxes in panels A and B summarise their distributions. Dashed lines mark HI = 1, CR = 10^−6^ and 10^−4^, and a Pb screening ratio of 1. The child and adult Pb ratios coincide because both use the same 200 mg kg^−1^ concentration comparator.

Under the R4 reference, the median composite-level child resident/public-access HI was 1.844 (composite P05–P95: 1.342–2.485), compared with 0.270 (0.187–0.357) for the adult resident (Table 4; Figure 4). Median composite cancer risks were 3.17 × 10^−5^ (1.88 × 10^−5^–4.28 × 10^−5^) for the child and 1.37 × 10^−5^ (8.40 × 10^−6^–1.87 × 10^−5^) for the adult. The R4 resident Pb screening ratio had a median of 0.493, and six of 26 composites (23.1%) had ratios ≥ 1. Adult-worker results were lower and are reported in Supplementary Table S6: median HI was 0.101 (0.078–0.138), median CR was 5.44 × 10^−6^ (3.99 × 10^−6^–8.98 × 10^−6^), and no worker composite exceeded HI = 1 or the Pb ratio of 1.

**Figure 5:**
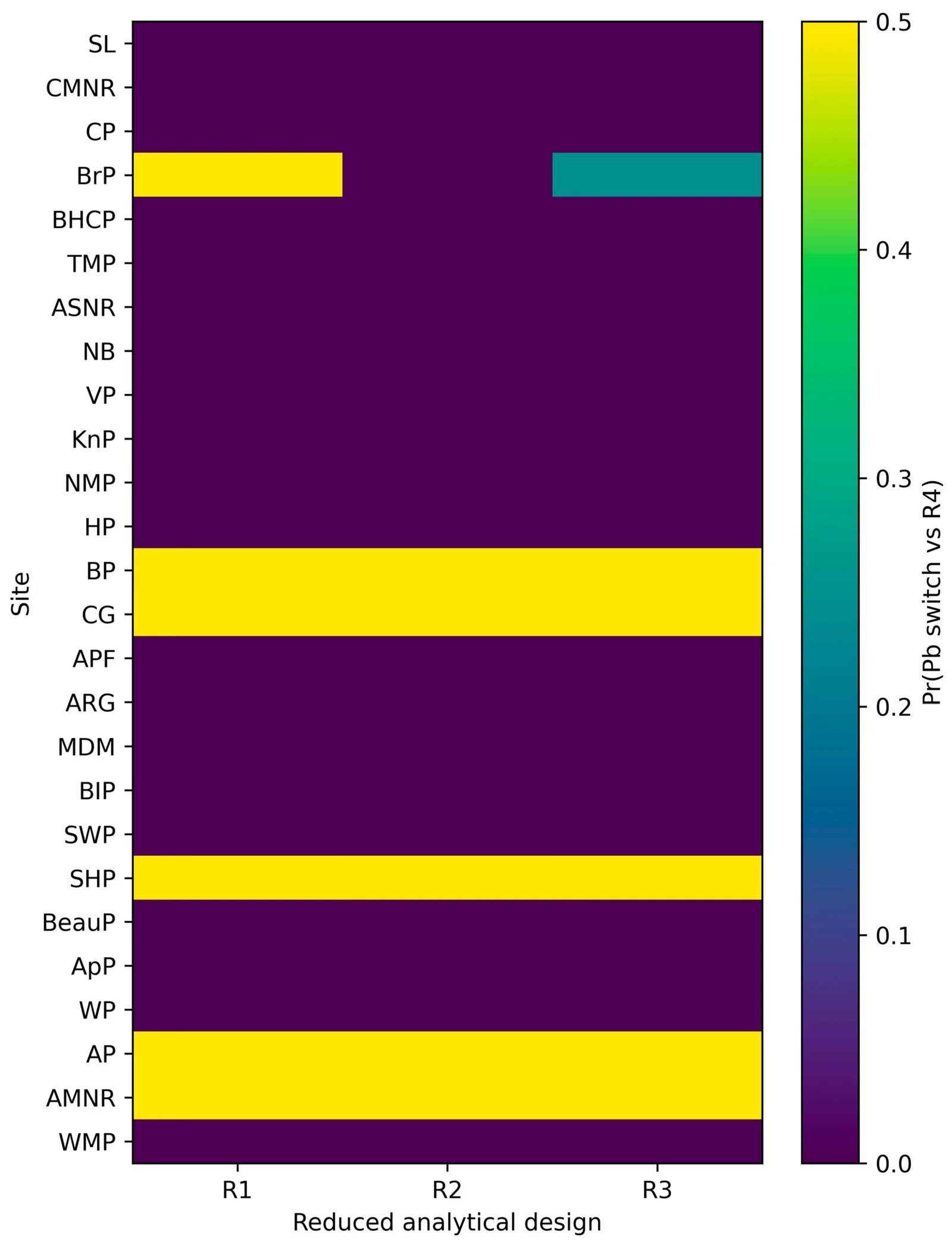
Composite-specific Pb screening-classification switch probabilities under reduced analytical designs relative to the full observed design (R4). Each cell shows the probability that the Pb screening classification for a given site-level composite under R1, R2, or R3 differed from its matched R4 classification across 10,000 simulations, using the 200 mg kg^−1^ resident/public-access Pb concentration comparator. Rows represent the 26 independent site-level composites, and columns represent the three reduced analytical designs. Child and adult resident/public-access switch probabilities were identical because both receptor scenarios used the same Pb concentration comparator and are therefore shown only once. No HI classification or cancer-risk-band switch occurred for any eligible receptor–composite combination.

No reduced design produced an HI classification switch or a cancer-risk-band switch relative to R4 in any receptor–composite combination across 10,000 simulations. In contrast, Pb screening was sensitive at composites close to the 200 mg kg^−1^ resident comparator. Mean composite-specific Pb switch probabilities were 9.57% for R1, 7.72% for R2, and 0.96% for R3; their maxima were 49.88%, 50.29%, and 24.88%, respectively (Figure 5). The same probabilities applied to the child and adult resident scenarios because Pb was classified using the same concentration ratio rather than receptor-specific dose equations.

The consequence of analytical design was therefore endpoint-dependent. Broad cumulative hazard and cancer-risk classifications were stable because their R4 values were not repeatedly crossing their respective decision boundaries. Pb classifications were less stable where full-design concentrations lay close to the screening threshold, and distributing determinations across both physical samples substantially reduced that instability. The principal CR driver was retained in every comparison; retention of the principal HI driver for the adult-resident scenario was 92.3% under R1, 94.2% under R2, and 100.0% under R3 (Supplementary Table S6).

## 4. Discussion

### 4.1. Environmental Heterogeneity Dominated the Measurement Hierarchy

The central variance result was consistent across detected and censored elements: most log-scale variability arose between composites. Between-composite proportions of 65.6–93.8% for fully detected elements, together with sensitivity estimates of 87.2% and 92.3% for Cd and Cr, show that differences among environmental units were larger than variation introduced at the physical-sample and analytical-subsample levels. This pattern is consistent with the broader principle that environmental heterogeneity and sampling can dominate the measurement process, while the relative contributions remain analyte-, scale-, and design-specific [3,4,5,6,7]. It also explains why reducing only analytical residual error had limited capacity to recover the R4 composite estimate when the selected physical portion differed from its companion sample.

The decomposition does not estimate all field-sampling uncertainty. Because the 850 increments were pooled before analysis, increment-to-increment variation within each sampling area cannot be separated from the resulting composite concentration. The between-composite term therefore represents differences among the 26 sampled areas, whereas the physical sample term represents residual heterogeneity between two portions withdrawn after homogenisation. The larger physical sample RPDs than analytical-pair RPDs indicate that physical subsampling contributed more lower-level variability than repeated analytical processing for most elements; they do not quantify the performance of the original field-sampling protocol.

Direct numerical comparison with field-duplicate studies would therefore be inappropriate because those studies target a different variance component. In a one-hectare agricultural field, de Zorzi et al. reported ratios of standard uncertainty from field sampling to total standard measurement uncertainty of 0.6–0.9 across Cr, Sc, and Zn and three sampling devices [29]. At two heterogeneous historic mining sites, Dołęgowska et al. reported element- and site-specific relative sampling uncertainties from 9.2% to 35.9%, depending on the element and statistical method [30]. By contrast, Buczko et al. found relative sampling uncertainties of approximately 10% in low-to-moderately heterogeneous arable fields and showed that total laboratory uncertainty could dominate when between-laboratory reproducibility was included [7]. Collectively, these findings support the context dependence of uncertainty allocation; they are not benchmarks for the 65.6–93.8% between-composite variance fractions reported here.

### 4.2. Analytical Replication Is Not Environmental Replication

The inferential-unit analysis provides an empirical demonstration of a general statistical principle [8]. The *N* = 102 calculation and the *N* = 26 calculation used the same underlying chemistry, yet their multiplicity-adjusted conclusions differed because only the latter used the composites as independent environmental units. Cluster-aware mixed models reached the same substantive conclusion as the composite-level analysis. The descriptive urban–rural patterns did not disappear; rather, their uncertainty was correctly tied to 18 urban and eight rural environmental units.

This distinction is especially important because the earlier geochemical characterisation of this dataset reported the *N* = 102 area-type contrasts [9]. The current methodological reanalysis deliberately reconstructs that analytical-record perspective and contrasts it with unit-corrected alternatives. Concentration summaries, sample identities, and directional patterns are unchanged. What changes is the evidential strength assigned to area-type differences after acknowledging nesting. Transparent cross-reference to the earlier article is therefore essential: the present work is not a second report of new concentrations, but an analysis of how hierarchical unit choice changes inference from an already characterised dataset.

Analytical duplicates remain scientifically valuable. They estimate repeatability, reveal discordant pairs, support quality assurance, and can improve a composite estimate. The significant a-versus-b label-direction offsets found here also warrant laboratory-record review. However, because the suffixes could not be verified as chronological order, this finding cannot establish drift or first-versus-second-run bias. More fundamentally, even perfect analytical repeatability would not increase the number of independent environmental composites.

### 4.3. Physical-Sample Coverage Generally Improved Performance at Comparable Analytical Effort

At similar analytical effort, R3 generally produced smaller concentration errors and more stable ranks than R2 on the principal summary measures. The reason follows directly from the hierarchy. R2 averaged duplicate determinations from one selected physical sample and therefore reduced analytical residual variability while retaining the selected sample’s physical-subsampling deviation. R3 used one determination from each physical sample and averaged across both sources of material, addressing a larger component of uncertainty. This interpretation is supported by both the variance partition and the paired-agreement summaries.

The practical implication is not that analytical duplicates are unnecessary, nor that two laboratory samples constitute two environmental sites. Instead, when the objective is to estimate the concentration of an already formed composite and the main lower-level uncertainty lies between physical portions, allocating a fixed number of determinations across portions can be more efficient than repeating one portion. Analytical duplicates may still be required for batch QA, method validation, censoring near the LoD, or diagnosis of anomalous results. A balanced design can therefore retain strategic analytical duplicates while prioritising physical sample coverage for estimation.

Evidence from field-scale contaminated-land investigations supports this broader allocation principle, although not a direct numerical comparison. Across six commercial site investigations, Boon et al. found that measurement uncertainty varied widely, and that field sampling dominated chemical analysis in at least five; their optimisation analyses consequently focused improvement on the dominant component [31]. The present R2–R3 comparison applies the same allocation logic at a lower, post-composite level: R3 improved coverage of two physical portions but did not add an independent field location or increase environmental *N*.

The extreme-pair analyses define the boundary of the claim. R3 continued to improve MdAPE and ±10% agreement after removing the named Ni, Cr, and Zn pairs, but Zn RMSE became essentially equal to or slightly worse than R2 in two sensitivities. RMSE gives disproportionate weight to large deviations and can respond differently from typical relative errors. Accordingly, the evidence supports general efficiency of R3 across the principal accuracy and decision metrics, not absolute superiority under every loss function.

### 4.4. Screening Consequences Were Endpoint- and Threshold-Dependent

Propagating design uncertainty through the previously defined exposure model showed substantial stability in the main dose-based classifications. No composite switched across HI = 1 or between the predefined cancer-risk bands. This does not mean that analytical design had no effect: continuous HIs and CRs varied, and principal HI drivers were less stable under the most reduced designs. Rather, the realised values remained on the same side of broad decision boundaries.

Pb provided a contrasting threshold experiment. Its screening check depends directly on concentration and was evaluated against a single resident/public-access comparator. Several composites lay sufficiently close to that threshold for selection of one physical sample to change the classification in approximately half of simulations. R2 provided little improvement over R1 in maximum Pb instability because both could repeatedly select the same unrepresentative physical portion. R3, which represented both portions, reduced the mean switch probability to 0.96% and the maximum to 24.88%.

Comparable threshold effects have been reported at the field-sampling scale. Ramsey and Argyraki estimated an uncertainty of approximately 50% at 95% confidence for Pb at one contaminated-land sampling site and proposed a probabilistic classification that made the potential for misclassification explicit [32]. Boon and Ramsey later showed for Cd that a site could appear non-problematic when assessed from uncertainty of the area mean, while individual locations remained possibly contaminated when uncertainty of individual measurements was considered [33]. Those studies concern field sampling rather than post-composite allocation, but the decision principle agrees with the present Pb analysis: classification instability is greatest when the plausible concentration range intersects a decision boundary.

These findings do not conflict with the below-unity group summaries reported previously [9]. Those summaries used concentrations aggregated across geographical groups, whereas the present decision-stability analysis classifies each composite separately. A group estimate or overall median below one can therefore coexist with individual composite ratios at or above the comparator.

This contrast illustrates why measurement uncertainty should be evaluated in the context of intended use [1,2,3,4]. A design adequate for preserving broad hazard or cancer-risk bands may be inadequate for a binary concentration exceedance near a regulatory or screening threshold. Conversely, a high relative concentration error may have no categorical effect when an endpoint lies far from its decision boundary. Reporting only analytical precision or only continuous risk estimates can therefore miss the decision-relevant consequence of hierarchical uncertainty.

### 4.5. Implications for Environmental Exposure-Study Design

Four design principles follow. First, the environmental unit must be defined before sample size or inferential tests are chosen. Second, replication should be placed at the hierarchical level responsible for the uncertainty that matters to the study objective. Third, reduced-design performance should be evaluated against decision-relevant endpoints, as well as concentration errors. Fourth, a full observed design is a reference configuration, not a substitute for an independently certified true value.

A prospective fully nested design would therefore comprise independent sites as the environmental replication level, multiple field replicates retained separately within each site, multiple independently withdrawn physical laboratory subsamples from each field replicate, and replicated analytical determinations of each physical subsample. Such a structure would permit between-site, within-site field, physical-subsampling, and analytical variability to be estimated separately. Where temporal variability is relevant to the intended inference, the complete hierarchy should additionally be repeated across independent sampling occasions.

For studies seeking generalisable environmental contrasts, additional independent sites or field sampling units are required; neither additional physical portions nor repeated instrumental measurements can replace them. Where temporal variability is relevant to the intended inference, the design should additionally include repeated sampling occasions so that spatial, temporal, physical-subsampling, and analytical components can be distinguished rather than conflated. For studies estimating composite concentration, distinct physical portions may provide greater return than duplicate determinations of one portion. For compliance or screening near thresholds, targeted replication around borderline samples and explicit classification-switch analysis may be particularly valuable. These principles extend beyond soil metal(loid)s to other particulate or heterogeneous environmental media in which field, preparation, and analytical levels are nested.

### 4.6. Limitations and Generalisability

Several limitations constrain interpretation. The study is a secondary analysis of one regional dataset, not a prospectively optimised replication experiment. Only two physical laboratory samples were available per composite, and two samples lacked analytical duplicates. Physical samples were withdrawn from an already pooled and homogenised composite; they are not independent field replicates. The original increments could not be analysed separately, so within-area field heterogeneity was not estimated. R4 uses all observed analytical information but remains an estimate, not truth, and simulated reduced designs inherit the structure and anomalies of the observed dataset. The 26 locations were not a probability sample of all soils in Leicester and Leicestershire, so the urban–rural contrasts should not be generalised beyond the sampled green spaces without additional sampling. Inferential precision was additionally limited by the 18-versus-8 site allocation: the conventional sensitivity calculation indicated 80% power only for a standardized between-group difference of approximately Cohen’s d = 1.24. Moderate area-type effects could therefore remain imprecisely estimated or undetected, and *q* > 0.05 should not be interpreted as evidence that the corresponding underlying effect is absent; effect sizes and their 95% confidence intervals provide the appropriate context for that uncertainty.

For Cd and Cr, the 500-realisation approach is a transparent sensitivity analysis rather than a fully likelihood-based censored mixed model. The proportions censored were modest and the main conclusion was stable, but future datasets with heavier censoring should use dedicated censored multilevel models. The a/b suffixes lacked verified chronology, limiting interpretation of signed offsets. A small number of extreme analytical pairs influenced residual-variance and RMSE metrics, although they did not reverse the main MdAPE or decision results.

Collision/reaction modes were unavailable during ICP-MS measurement; sample-specific polyatomic interference therefore could not be excluded fully for ^52^Cr and, to a lesser extent, ^75^As and ^51^V. This chemical limitation is separate from the hierarchical-design analysis, but it limits interpretation of element-specific concentrations. Cr-specific toxicity endpoints were additionally precluded by the absence of oxidation-state speciation.

The simulations isolate only one component of total uncertainty. Hazard, exposure, and risk assessment also contain uncertainty arising from exposure parameters, toxicity values, model structure, and conceptual assumptions; Ramsey [34] emphasised the need to distinguish these sources and propagate those that can be quantified. Receptor and toxicity inputs were intentionally held fixed here. The reported percentile ranges and switch probabilities therefore characterise sensitivity to concentration representation, not total uncertainty in dose or health risk.

The exposure calculations are precautionary screening estimates carried forward unchanged from the prior study [9]. The HNO_3_/HCl digestion generated acid-recoverable or pseudo-total concentrations rather than complete dissolution of resistant silicates; composite sampling can conceal local hotspots; bioaccessibility was not measured; total As was treated as inorganic; Cr-specific toxicity was excluded because speciation was unavailable; and Pb ratios are screening comparisons rather than blood-lead predictions. The stability results apply to this fixed model and these decision thresholds and should not be confused with validation of the toxicological assumptions or with site-specific risk assessment.

## 5. Conclusions

In this hierarchical topsoil dataset, between-composite heterogeneity was the dominant source of log-scale variability, whereas analytical repeatability was generally high. At approximately two determinations per composite, distributing analytical effort across the two available physical laboratory samples generally improved concentration accuracy and rank stability relative to duplicating the analysis of a single physical sample. Treating 102 nested analytical records as independent environmental observations generated FDR-significant urban–rural signals that did not remain FDR-significant in analyses based on the 26 independent composites or in cluster-aware models. The downstream consequences of reduced replication were decision-specific: HI classifications and cancer-risk bands remained stable, whereas Pb classifications became sensitive to sample allocation when concentrations approached the screening threshold. These findings show that replication should be allocated at the hierarchical level relevant to both the intended inferential unit and the decision being supported. Accordingly, future studies should reduce decision-relevant uncertainty by allocating replication to the level at which it arises, prioritising independent field units for environmental inference and repeated sampling occasions when temporal variability is also of interest. Analytical replication improves measurement precision, but it does not create environmental replication.

## Figures and Tables

**Table 1:** Hierarchical study structure and simulated analytical designs.

**Observed hierarchical structure**					
**Level**	**Count**	**Nesting**	**Independent Environmental Unit?**	**Role**	**Interpretive Constraint**
Field increments	850	Pooled within 26 composites	No	Form each composite	Increment variance unavailable after pooling
Site-level composites	26	18 urban; 8 rural	Yes	Independent units for environmental inference	Defensible environmental *N* = 26
Physical laboratory samples	52	Two per composite	No	Represent physical subsampling	Nested within parent composite
Analytical determinations	102	50 samples duplicated; 2 with one result	No	Represent analytical variability	Do not create environmental replication
**Reduced analytical designs evaluated against the full observed design**					
**Design**	**Physical Samples/Composite**	**Determinations Selected**	**Composite Estimator**	**Expected Determinations per Composite**	**Status**
R1	1	One from selected sample	Selected determination	1.000	Minimal design
R2	1	All valid from selected sample	Mean within one sample	1.962	Analytical replication only
R3	2	One from each sample	Equal-weight mean	2.000	Physical-sample coverage prioritised
R4	2	All valid from both samples	Mean within sample, then mean of sample means	3.923	Full-design reference

**Table 2:** Variance partitioning and reproducibility across hierarchical levels.

Element	Between Composite (%)	Between Physical Sample (%)	Analytical Residual (%)	Composite ICC [Interval]	Analytical RPD (%)	Physical Sample RPD (%)
As	69.0	24.8	6.3	0.690 [0.374–0.917]	3.7	9.0
Be	92.1	6.5	1.3	0.921 [0.812–0.969]	4.6	7.2
Cd	87.2	7.9	4.7	0.872 [0.830–0.896]	5.3	9.6
Co	86.1	11.9	2.0	0.861 [0.701–0.945]	4.2	6.0
Cr	92.3	5.4	2.3	0.923 [0.906–0.931]	3.2	8.7
Cu	93.8	5.2	1.0	0.938 [0.891–0.970]	5.3	10.6
Fe	79.0	20.5	0.4	0.790 [0.599–0.899]	0.6	6.0
Mn	65.6	31.6	2.8	0.656 [0.272–0.895]	3.3	9.2
Ni	72.3	20.5	7.1	0.723 [0.433–0.936]	4.6	10.4
Pb	89.5	10.3	0.3	0.895 [0.780–0.969]	3.8	8.1
V	87.1	10.9	2.0	0.871 [0.659–0.963]	3.4	6.1
Zn	79.5	8.8	11.6	0.795 [0.607–0.931]	4.2	9.5

For the 10 fully detected elements, percentages and ICCs were estimated from log-scale mixed models; intervals are 95% site-cluster bootstrap confidence intervals (1000 resamples). For Cd and Cr, values are medians across 500 censor-compatible realisations without LoD/2 substitution; bracketed limits are 2.5th–97.5th realisation percentiles, not bootstrap confidence intervals. Analytical RPDs use 50 paired samples; physical sample RPDs use 26 pairs. The chronology of a/b analytical labels was not confirmed. RPD, relative percent difference; ICC, intraclass correlation coefficient.

**Table 3:** Efficiency of alternative analytical designs relative to the full observed design.

Design	Expected Determinations per Composite	Median Element MdAPE (%)	Mean *P* (Within ±10% of R4)	Mean Spearman *ρ*	Mean Absolute Rank Displacement
R1	1.00	4.91	74.3%	0.948	1.50
R2	1.96	4.27	77.5%	0.953	1.42
R3	2.00	1.32	96.7%	0.991	0.47
R4	3.92	0.00	100.0%	1.000	0.00

Values summarise 12 analytes over 10,000 simulations. MdAPE is the median of element-specific median absolute percentage errors; remaining performance quantities are means across elements. R2 and R3 used approximately two determinations per composite, but R3 distributed them across both physical samples. The small R2 effort deficit reflects the two single-determination Brocks Hill samples. Leave-one-extreme-pair-out sensitivity did not support universal R3 superiority across every metric, particularly Zn RMSE.

**Table 4:** Propagation of analytical-design uncertainty into exposure-screening decisions.

**Full-design (R4) resident/public-access screening summaries**						
**Receptor**	***n***	**Median HI [P05–P95]**	**Sites with HI ≥ 1, n (%)**	**Median CR [P05–P95]**	**Median Pb Ratio**	**Sites with Pb Ratio ≥ 1, n (%)**
Child	26	1.844 [1.342–2.485]	26 (100%)	3.17 × 10^−5^ [1.88 × 10^−5^–4.28 × 10^−5^]	0.493	6 (23.1%)
Adult	26	0.270 [0.187–0.357]	0 (0%)	1.37 × 10^−5^ [8.40 × 10^−6^–1.87 × 10^−5^]	0.493	6 (23.1%)
**Classification-switch probabilities for reduced designs versus R4**						
**Design**	**Mean HI Switch**	**Maximum HI Switch**	**Mean CR-Band Switch**	**Maximum CR-Band Switch**	**Mean Pb Switch**	**Maximum Pb Switch**
R1	0%	0%	0%	0%	9.57%	49.88%
R2	0%	0%	0%	0%	7.72%	50.29%
R3	0%	0%	0%	0%	0.96%	24.88%

Pb was evaluated as concentration/200 mg kg^−1^ for resident/public-access scenarios rather than as an RfD-based HI. Total Cr was excluded from Cr-specific HQ, HI and CR because speciation was unavailable. Adult-worker outputs (18 urban sites; Pb/800 mg kg^−1^) are supplementary. HI, hazard index; CR, cumulative cancer risk; P05/P95, fifth/95th site percentiles.

## Data Availability

The data and reproducibility materials supporting this study are publicly available in Figshare at https://doi.org/10.6084/m9.figshare.33985933. The repository contains the final analytical datasets, field-sampling and spatial data, R code, publication tables and figures, Supplementary Materials, and quality-assurance/provenance documentation required to reproduce the reported analyses. The datasets are released under the Creative Commons Attribution 4.0 International licence (CC BY 4.0).

## Supplementary Materials

The following supporting information can be downloaded at https://www.mdpi.com/article/10.3390/toxics14100887/s1, Table S1: Sampling locations, site characteristics and spatial coverage of the field-sampling design; Table S2: Analytical quality-control and agreement diagnostics; Table S3: Element-specific performance of R2 versus R3 and sensitivity to four extreme analytical pairs; Table S4: Urban–rural inference under alternative definitions of the inferential unit; Table S5: Exposure and toxicity inputs used in the screening calculations; Table S6: Adult-worker screening summary and stability of the principal HI and CR drivers; Figure S1: Relative percent difference distributions for paired analytical determinations and physical sample estimates; Figure S2: Bland–Altman plots for paired analytical determinations; Figure S3: Composite-rank concordance under reduced analytical designs; Figure S4: Sensitivity of the Cd and Cr variance decomposition to left-censored observations; Figure S5: Urban–rural effect sizes and 95% confidence intervals under unit-corrected inferential approaches.
