# Supplementary material for: Analytical Replication Improves Precision but Does Not Create Environmental Replication: Hierarchical Uncertainty and Decision Stability in Metal(loid) Soil Exposure Screening
Source: Toxics. 2026 Oct 2;14(10):887. doi: 10.3390/toxics14100887 (PMC13709735; doi:10.3390/toxics14100887)
Supplement: Supplementary file 1 [file toxics-14-00887-s001.zip › Supplementary Figures_revised.pdf]

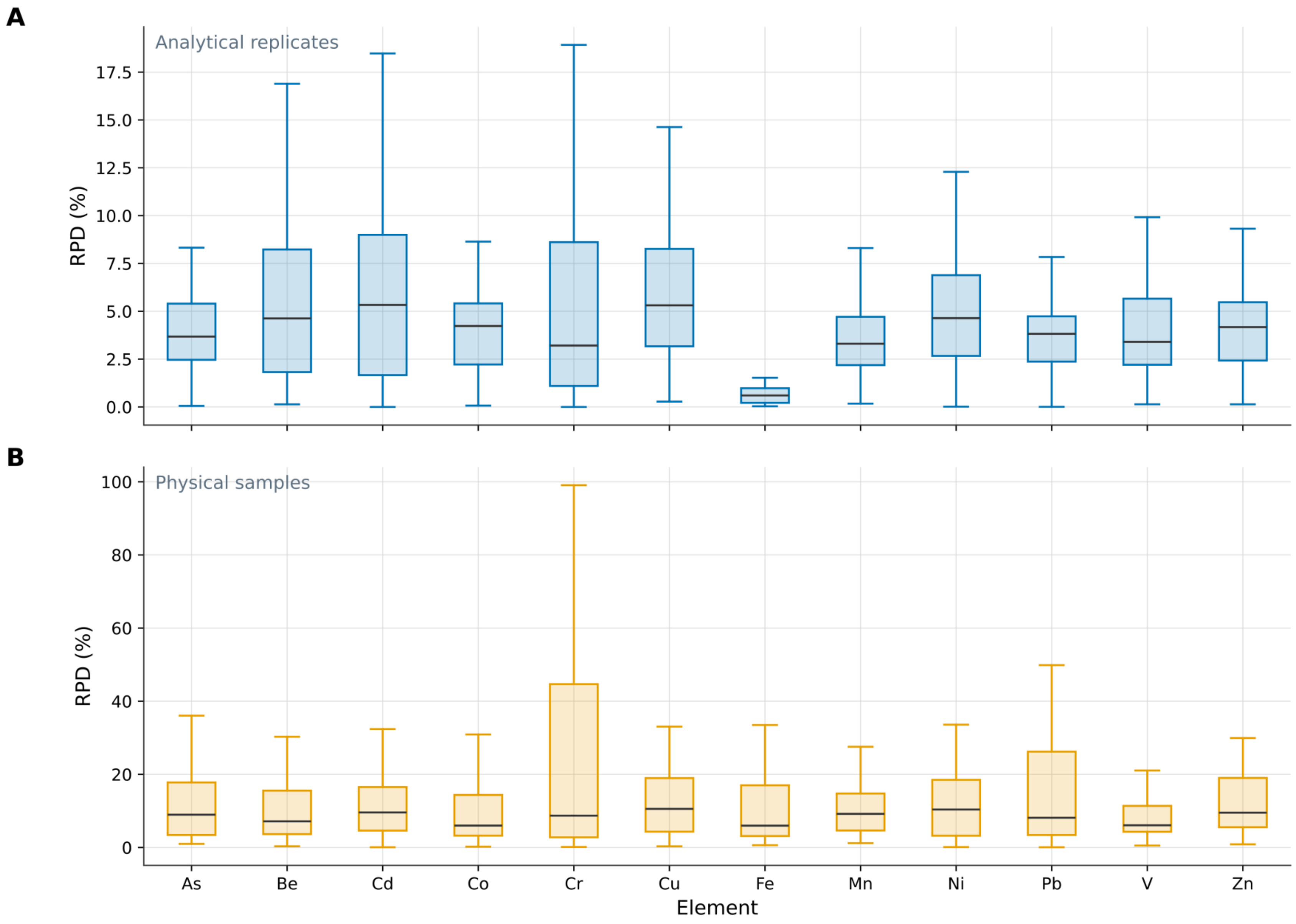

Figure S1. Relative percent difference (RPD) distributions for paired analytical determinations within physical samples (A) and paired physical-sample estimates within composites (B), shown by element. Boxes denote medians and interquartile ranges; whiskers extend to the most extreme observations within 1.5 times the interquartile range. Outliers are not drawn, and the vertical scales differ between panels.

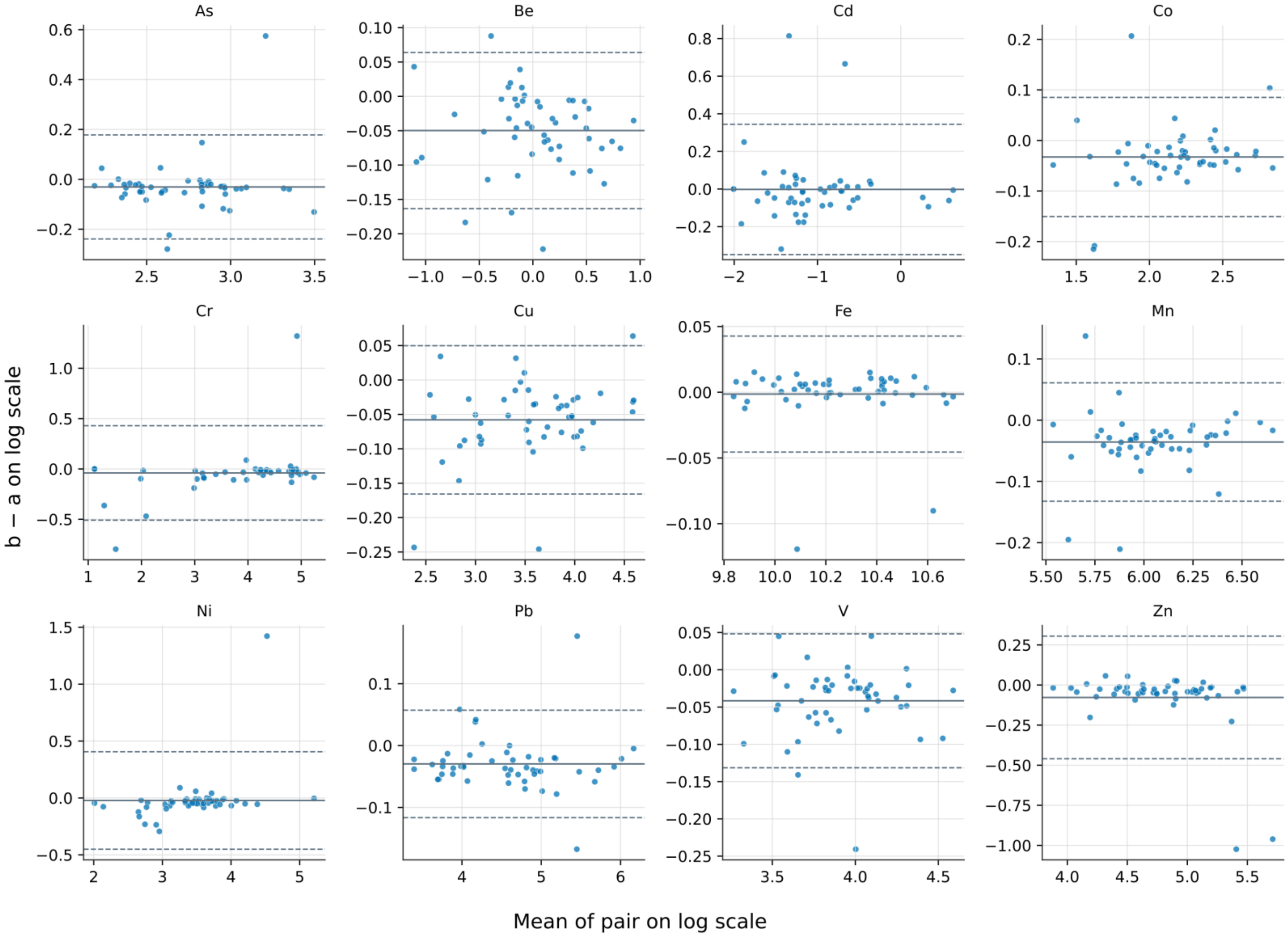

Figure S2. Bland–Altman plots for paired analytical determinations across the 12 elements. The horizontal axis is the pair mean on the log scale and the vertical axis is the b-minus-a log difference. Solid horizontal lines show the mean difference and dashed lines show the 95% limits of agreement (mean difference ± 1.96 standard deviations). The a/b suffixes indicate label direction only; verified analytical chronology was unavailable.

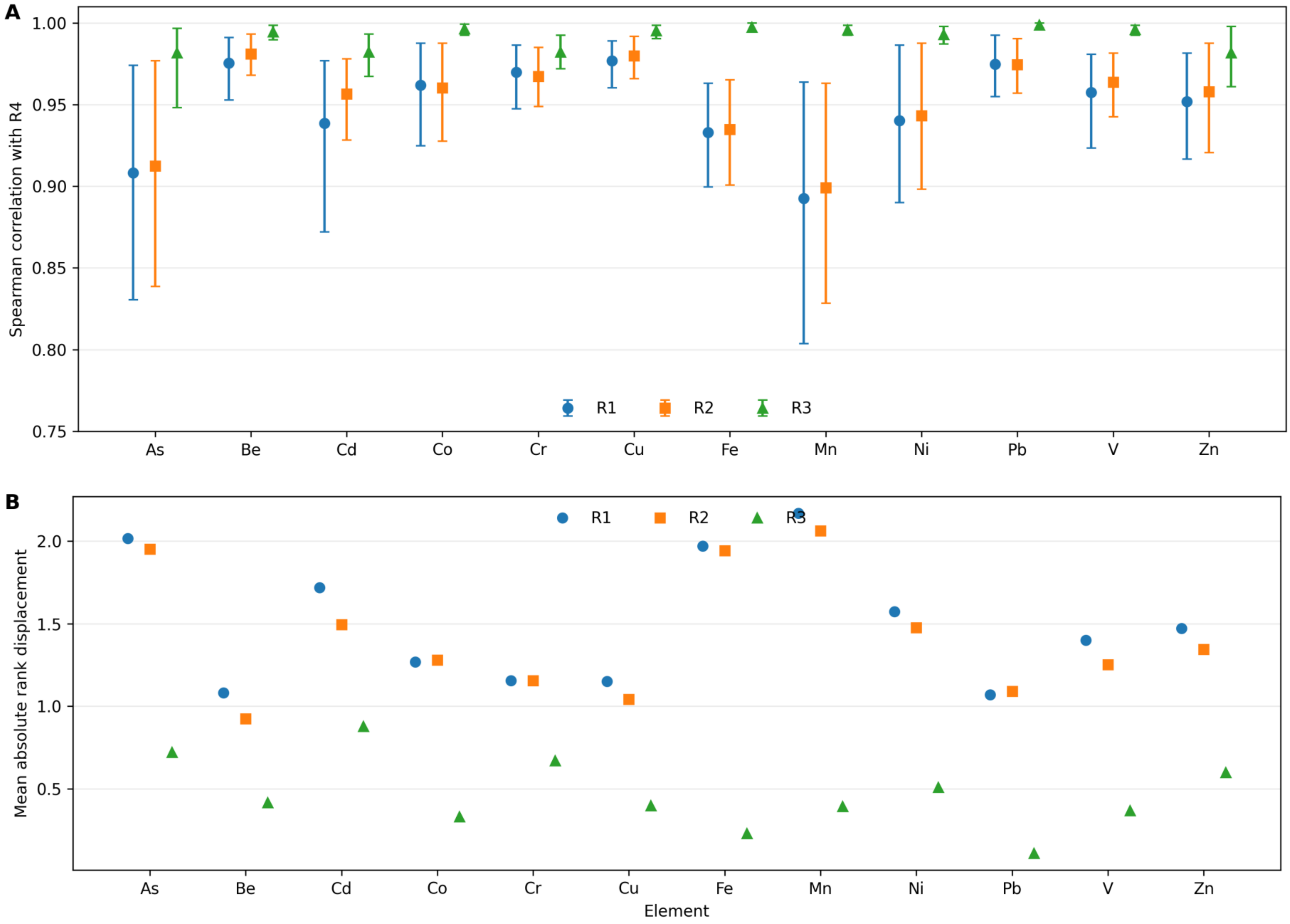

Figure S3. Composite-rank concordance under reduced analytical designs relative to R4 using the final POST-v1.4 simulation outputs. Panel A shows the mean Spearman rank correlation, with 2.5th–97.5th percentile intervals across 10,000 matched iterations. Panel B shows mean absolute rank displacement. Symbols identify R1, R2 and R3.

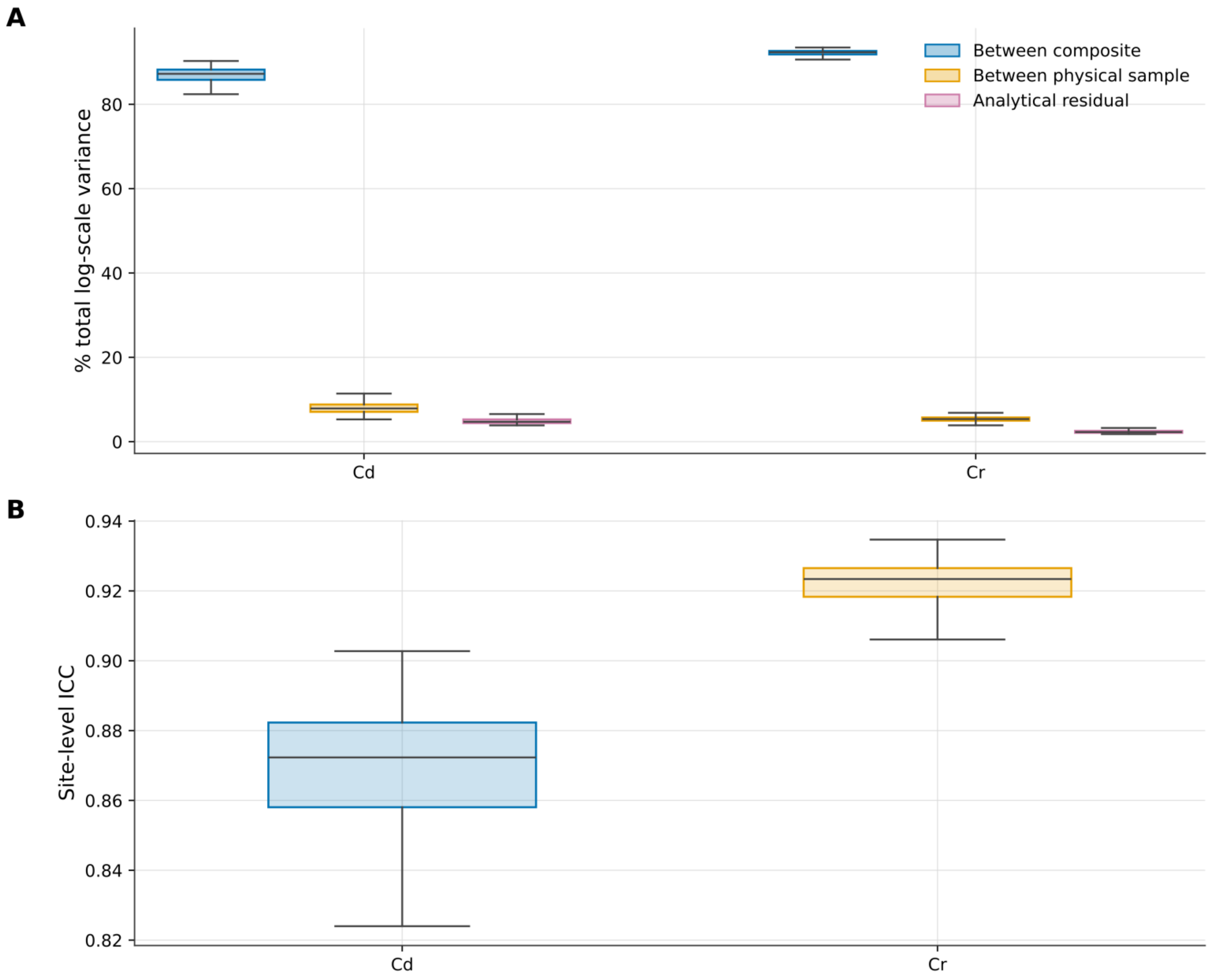

Figure S4. Sensitivity of the Cd and Cr variance decomposition to left-censored observations. Panel A shows the distributions of the percentage of total log-scale variance assigned to the between-composite, between-physical-sample and analytical-residual components across 500 censor-compatible realisations. Panel B shows the corresponding composite-level intraclass correlation coefficients. These distributions quantify sensitivity to plausible values below the limit of detection, not sampling confidence intervals.

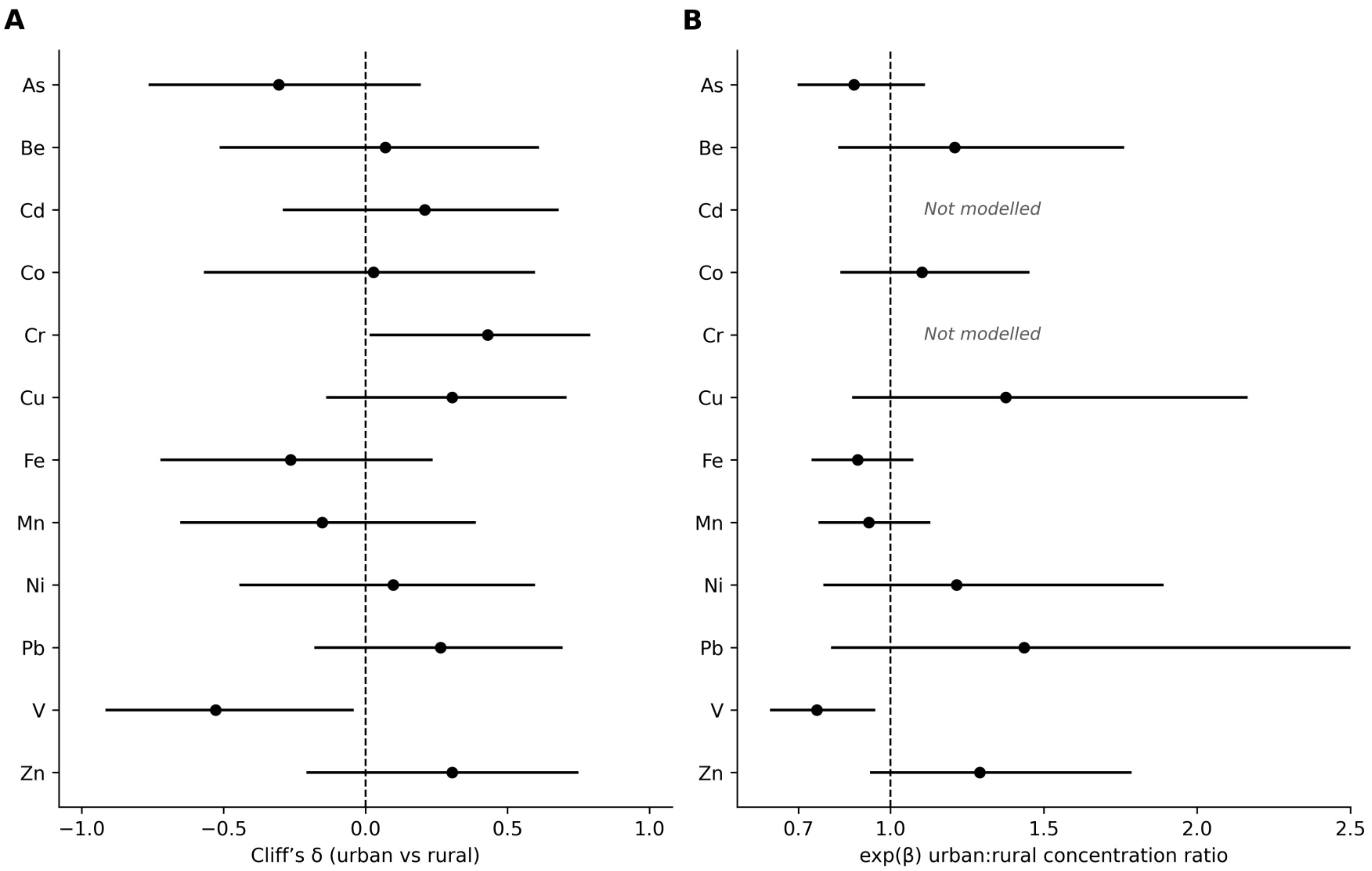

Points = estimates; horizontal bars = 95% CI. Cd and Cr were not modelled in panel B because mixed models were restricted to the 10 fully detected elements.

Figure S5. Urban–rural effect sizes under unit-corrected inferential approaches. Panel A shows composite-level Cliff's δ (urban versus rural) with stratified-bootstrap 95% confidence intervals for the 26 independent site-level composites. Panel B shows the exponentiated fixed effect, exp(β), from the cluster-aware log-scale mixed model, interpreted as the estimated urban:rural concentration ratio, with profile-likelihood 95% confidence intervals. Dashed vertical reference lines indicate $\delta = 0$ and ratio = 1, respectively. Cd and Cr were not modelled in Panel B because they were left-censored. Full raw p and Benjamini–Hochberg-adjusted q values are reported in Table S4.
