# Supplementary material for: Analytical Replication Improves Precision but Does Not Create Environmental Replication: Hierarchical Uncertainty and Decision Stability in Metal(loid) Soil Exposure Screening
Source: Toxics. 2026 Oct 2;14(10):887. doi: 10.3390/toxics14100887 (PMC13709735; doi:10.3390/toxics14100887)
Supplement: Supplementary file 1 [file toxics-14-00887-s001.zip › Supplementary Tables_revised.pdf]

Table S1. Sampling locations, site characteristics and spatial coverage of the field-sampling design.

Panel A. Site characteristics and georeferenced sampling locations

Site code	Site name	Area type	Quadrant	Documented park/ reserve area (ha)	Latitude (WGS84)	Longitude (WGS84)	Field increments, n
CG	Castle Gardens	Urban	NW	—	52.63332	-1.14124	25
AP	Abbey Park	Urban	NE	—	52.64921	-1.13693	25
VP	Victoria Park	Urban	SE	28.0	52.62274	-1.11755	25
KnP	Knighton Park	Urban	SE	31.5	52.60166	-1.10979	25
BrP	Braunstone Park	Urban	SW	68.0	52.62467	-1.17211	25
WP	Western Park	Urban	NW	72.0	52.63942	-1.18260	25
BeauP	Beaumont Park	Urban	NW	—	52.66668	-1.16267	25
AppP	Appleton Park	Urban	NE	—	52.66112	-1.09728	25
SHP	Spinney Hill Park	Urban	NE	13.8	52.63438	-1.10811	25
AMNR	Aylestone Meadows Nature Reserve	Urban	SW	—	52.60935	-1.15870	25
MM	Mossdale Meadows	Urban	SW	9.51	52.61204	-1.18430	25
SWP	Stokes Wood Park	Urban	NW	—	52.64500	-1.16017	25
BP	Bede Park	Urban	SW	—	52.62919	-1.14587	25
NMP	Nelson Mandela Park	Urban	SE	—	52.62581	-1.13107	25
WMP	Watermead Park	Urban	NE	137.5	52.67015	-1.11184	25
HP	Humberstone Park	Urban	NE	—	52.64003	-1.08472	25
APF	Aylestone Playing Fields	Urban	SW	—	52.60203	-1.17127	25
ARG	Aylestone Recreation Ground	Urban	SE	—	52.60795	-1.13347	25
BdP	Bradgate Park	Rural	NW	336.0	52.68564	-1.21476	50
CMNR	Cossington Meadows Nature Reserve	Rural	NE	88.9	52.71903	-1.11288	50
ASNR	Alterstones Nature Reserve	Rural	NW	3.70	52.69371	-1.28427	50
SL	Syston Lakes	Rural	NE	9.50	52.70048	-1.09340	50
TMP	The Miles Piece	Rural	NE	0.70	52.65761	-1.00804	50
BHCP	Brocks Hill Country Park	Rural	SE	30.0	52.58932	-1.08544	50
NB	Narborough Bog	Rural	SW	9.20	52.57620	-1.19080	50
CP	Croft Pasture	Rural	SW	5.80	52.55775	-1.24990	50

Park/reserve areas are the approximate whole-site areas recorded numerically in the original study site descriptions; they are distinct from the horizontal sampling footprint quantified in Panel B. Coordinates are WGS84 site-level sampling/reference coordinates used for cartographic presentation.

Panel B. Horizontal spatial coverage of the field-sampling design

Sampling unit	Field increments, n	Field sampling geometry	Georeferenced increments, *n*	Approx. horizontal sampling footprint (ha)	Maximum sampled extent (m)
Urban sites (n = 18)	25 per site	25-point cross; 2 m spacing	1 starting GPS position per site*	0.0288	24
BdP — Bradgate Park	50	Spatially distributed / random	50	26.46	1064
CMNR — Cossington Meadows Nature Reserve	50	Spatially distributed / random	50	36.09	2361
ASNR — Alterstones Nature Reserve	50	Spatially distributed / random	50	12.56	1053

Sampling unit	Field increments, n	Field sampling geometry	Georeferenced increments, *n*	Approx. horizontal sampling footprint (ha)	Maximum sampled extent (m)
SL — Syston Lakes	50	Spatially distributed / random	50	2.11	241
TMP — The Miles Piece	50	Spatially distributed / random	50	0.60	99
BHCP — Brocks Hill Country Park	50	Spatially distributed / random	50	2.68	323
NB — Narborough Bog	50	Spatially distributed / random	50	4.46	312
CP — Croft Pasture	50	Spatially distributed / random	50	3.91	290

For the urban design, the original protocol used a central starting point with six additional increments at 2 m intervals along each of the north, south, east and west arms (25 increments in total). The resulting nominal convex-hull footprint is 288 m^2 (0.0288 ha), with a maximum north–south/east–west extent of 24 m. For rural/semi-rural sites, the sampling footprint was calculated from the convex hull of the 50 original field GPS positions after projection to British National Grid (EPSG:27700); maximum sampled extent is the largest pairwise distance between recorded field points. *Nelson Mandela Park uses the same prescribed urban field design; its map location is represented by the park reference coordinate used in Figure 1.

Table S2. Analytical quality-control and agreement diagnostics.

Panel A. Element-specific limits of detection, censoring and reference-material recoveries

Element	LoD (mg kg⁻¹)	Detected, n/N (%)	Left-censored, n/N (%)	CRM059 recovery (%)	NCS DC87101 recovery (%)
As	0.394507	102/102 (100.0%)	0/102 (0.0%)	106.3	104.6
Be	0.0650652	102/102 (100.0%)	0/102 (0.0%)	102.8	70.4
Cd	0.161859	96/102 (94.1%)	6/102 (5.9%)	105.2	61.2*
Co	0.153038	102/102 (100.0%)	0/102 (0.0%)	108.6	100.3
Cr	3.68347	94/102 (92.2%)	8/102 (7.8%)	106.9	87.1
Cu	1.33475	102/102 (100.0%)	0/102 (0.0%)	106.3	102.5
Fe	4.39998	102/102 (100.0%)	0/102 (0.0%)	107.6	NR†
Mn	1.65753	102/102 (100.0%)	0/102 (0.0%)	94.9	100.5‡
Ni	5.64050	102/102 (100.0%)	0/102 (0.0%)	102.1	108.0
Pb	0.697626	102/102 (100.0%)	0/102 (0.0%)	99.2	74.2
V	0.137315	102/102 (100.0%)	0/102 (0.0%)	103.5	90.4
Zn	1.32161	102/102 (100.0%)	0/102 (0.0%)	104.6	33.7

LoD was calculated as three times the standard deviation of nine procedural digestion blanks. Results at or below the LoD were classified as left-censored. Recovery was calculated as 100 × measured concentration/assigned concentration. CRM059 Trace Metals–Loamy Clay 2, lot LRAA8361, was the principal certified reference material; NCS DC87101 Soil was a supplementary check. *The assigned Cd concentration in NCS DC87101 was reference-only. †NCS DC87101 assigns Fe as Fe_2O_3 (4.69%) and FeO (approximately 0.34%); because the study reports elemental Fe, an elemental-Fe recovery was not calculated from these oxide assignments. Fe was quantified directly as elemental Fe by AAS. ‡Mn recovery was calculated after conversion from MnO.

Panel B. Agreement at the analytical-determination and physical-sample levels

Element	Analytical pairs, n	Analytical median RPD (%)	Analytical P95 RPD (%)	Analytical CCC (log)	Analytical BH-*q*	Physical pairs, *n*	Physical median RPD (%)	Physical P95 RPD (%)	Physical CCC (log)	Physical BH-*q*
As	50	3.68	18.88	0.936	2.89e-06	26	8.97	39.87	0.700	0.8
Be	50	4.63	15.01	0.986	5.22e-07	26	7.15	29.22	0.925	0.329
Cd	50	5.33	28.59	0.958	0.0407	26	9.58	61.78	0.894	0.647
Co	50	4.23	15.98	0.980	5.7e-06	26	5.99	29.62	0.865	0.8
Cr	50	3.21	41.54	0.980	4.03e-06	26	8.71	90.41	0.928	0.572
Cu	50	5.31	13.41	0.990	2.51e-07	26	10.56	41.31	0.941	0.253
Fe	50	0.60	1.51	0.995	0.0407	26	5.97	32.65	0.788	0.573
Mn	50	3.30	12.97	0.972	4.89e-07	26	9.19	38.13	0.655	0.8
Ni	50	4.64	23.28	0.929	4.57e-06	26	10.38	90.89	0.731	0.8
Pb	50	3.82	7.63	0.997	1.14e-06	26	8.13	74.58	0.898	0.0138

Element	Analytical pairs, n	Analytical median RPD (%)	Analytical P95 RPD (%)	Analytical CCC (log)	Analytical BH-*q*	Physical pairs, *n*	Physical median RPD (%)	Physical P95 RPD (%)	Physical CCC (log)	Physical BH-*q*
V	50	3.40	10.51	0.978	2.51e-07	26	6.07	24.04	0.875	0.329
Zn	50	4.17	21.53	0.884	5.22e-07	26	9.52	60.68	0.815	0.572

RPD, relative percent difference; P95, 95th percentile; CCC, Lin concordance correlation coefficient on the log scale; BH-*q*, Benjamini–Hochberg-adjusted paired Wilcoxon *p*-value within each nesting level. For Cd and Cr, paired descriptive statistics used the conditional-median working values defined in Section 2.4. Analytical a/b labels identify label direction only because chronology was unavailable; these tests assess label-direction offsets, not replicate-order bias. Physical comparisons use laboratory sample 2 versus sample 1 within each composite.

Table S3. Element-specific performance of R2 versus R3 and sensitivity to four extreme analytical pairs.

Panel A. Element-specific performance relative to the full observed design (R4)

Element	R2 relative RMSE (%)	R3 relative RMSE (%)	R2 MdAPE (%)	R3 MdAPE (%)	R2 within ±10%	R3 within ±10%	R2 mean Spearman ρ	R3 mean Spearman ρ	R2 mean rank displacement	R3 mean rank displacement
As	11.21	4.15	4.48	1.25	80.8%	96.2%	0.913	0.982	1.95	0.72
Be	8.63	2.66	3.58	1.58	80.8%	100.0%	0.981	0.995	0.92	0.41
Cd	13.66	7.11	4.78	2.07	79.6%	89.6%	0.957	0.982	1.49	0.88
Co	8.53	2.41	3.00	1.29	80.8%	98.1%	0.960	0.996	1.28	0.33
Cr	21.46	10.36	3.59	1.26	65.4%	87.7%	0.967	0.982	1.16	0.67
Cu	9.40	2.74	5.28	1.65	76.9%	100.0%	0.980	0.995	1.04	0.40
Fe	7.60	0.76	2.99	0.17	76.9%	100.0%	0.935	0.997	1.94	0.23
Mn	10.15	2.12	4.60	0.96	84.6%	100.0%	0.899	0.996	2.06	0.39
Ni	18.69	9.00	5.19	1.36	76.9%	96.2%	0.943	0.993	1.48	0.51
Pb	15.37	1.84	4.06	1.07	61.5%	100.0%	0.974	0.999	1.09	0.11
V	7.33	2.17	3.04	1.46	88.5%	100.0%	0.964	0.996	1.25	0.37
Zn	12.42	8.77	4.76	1.47	76.9%	92.3%	0.958	0.982	1.34	0.60

Metrics were calculated across 10,000 matched POST-v1.4 iterations. R2 averaged all valid determinations from one selected physical sample (expected effort, 1.962 determinations per composite); R3 averaged one determination from each of the two physical samples (2.000 determinations per composite). MdAPE denotes median absolute percentage error.

Panel B. Leave-one-extreme-pair-out sensitivity of the R2–R3 concentration comparison

Excluded pair (element/sample)	Site	Pair RPD (%)	R2 relative RMSE (%)	R3 relative RMSE (%)	R2 within ±10%	R3 within ±10%
Ni / 3760	SL	122.3	17.01	2.82	80.0%	100.0%
Cr / 3760	SL	115.7	20.39	6.82	68.0%	91.2%
Zn / 3759	SL	94.3	11.02	6.44	80.0%	96.0%
Zn / 3819	BP	89.3	10.47	6.58	80.0%	96.0%

Each sensitivity analysis retained 25 composites. Exact removal of both determinations made the two-physical-sample estimate undefined for the affected site–element, so that site–element was omitted symmetrically from R2, R3 and R4 for the corresponding sensitivity analysis only. The four excluded extreme-pair sites are at Syston Lakes (SL) or Braunstone Park (BP), whereas the singleton implementation fix affects BHCP only. POST-v1.4 relative RMSE and within-±10% sensitivity were updated exactly from additive squared-error and success counts. Leave-one-out MdAPE is intentionally not reported after the singleton fix because a median cannot be reconstructed exactly from archived aggregate summaries without mixing analytical states.

Table S4. Urban–rural inference under alternative definitions of the inferential unit.

Panel A. Deliberately naïve N = 102 analytical-record comparison (included only to demonstrate pseudoreplication)

Element	Cliff's δ Urban vs rural	Raw p	BH-q
As	-0.306	0.0154	0.0369
Be	0.098	0.436	0.476
Cd	0.221	0.0798	0.12
Co	0.058	0.644	0.644
Cr	0.440	0.000478	0.00287
Cu	0.360	0.00428	0.0171
Fe	-0.256	0.0419	0.0838
Mn	-0.119	0.343	0.412
Ni	0.210	0.0955	0.127
Pb	0.227	0.072	0.12
V	-0.471	0.000185	0.00222
Zn	0.332	0.00837	0.0251

Panel B. Composite-level comparison using the 26 independent site-level estimates

Element	Urban median	Rural median	Cliff's δ	95% CI	Raw p	BH-q
As	14.491	18.540	-0.306	-0.764 to 0.194	0.222	0.499
Be	1.010	1.147	0.069	-0.514 to 0.611	0.781	0.852
Cd	0.310	0.303	0.208	-0.292 to 0.681	0.405	0.607
Co	8.968	10.427	0.028	-0.569 to 0.597	0.912	0.912
Cr	85.538	36.561	0.431	0.014 to 0.792	0.085	0.499
Cu	44.870	33.554	0.306	-0.139 to 0.708	0.222	0.499
Fe	26698.750	33182.500	-0.264	-0.722 to 0.236	0.291	0.499
Mn	413.594	450.283	-0.153	-0.653 to 0.389	0.541	0.722
Ni	34.679	33.578	0.097	-0.444 to 0.597	0.697	0.837
Pb	103.818	57.431	0.264	-0.181 to 0.694	0.291	0.499
V	47.179	64.006	-0.528	-0.917 to -0.042	0.0348	0.417
Zn	128.068	107.559	0.306	-0.208 to 0.750	0.222	0.499

Panel C. Cluster-aware log-scale mixed models for the ten fully detected elements

Element	Urban:rural ratio, exp(β)	95% CI	Raw *p*	BH-*q*
As	0.881	0.697 to 1.112	0.273	0.443
Be	1.209	0.830 to 1.762	0.31	0.443
Cd	Not modelled	—	—	—
Co	1.103	0.836 to 1.453	0.474	0.474
Cr	Not modelled	—	—	—
Cu	1.376	0.874 to 2.164	0.16	0.441
Fe	0.893	0.742 to 1.075	0.22	0.441
Mn	0.929	0.765 to 1.130	0.448	0.474
Ni	1.215	0.781 to 1.891	0.373	0.467
Pb	1.435	0.806 to 2.556	0.209	0.441
V	0.759	0.607 to 0.950	0.0181	0.181
Zn	1.291	0.933 to 1.786	0.118	0.441

Cliff's δ is oriented as urban versus rural. Composite-level 95% confidence intervals were obtained by stratified nonparametric bootstrap resampling of sites within area type (10,000 replicates). Cluster-aware models used the nested analytical records from 26 site clusters; exp(β) is the estimated Urban:Rural concentration ratio and 95% confidence intervals are profile-likelihood intervals. Cd and Cr were not modelled in Panel C because they were left-censored. BH-*q* values were adjusted across elements within each inferential approach. The N=102 analysis is deliberately invalid for environmental inference and is retained only to demonstrate the consequence of treating nested analytical records as independent environmental observations.

Table S5. *Exposure and toxicity inputs used in the screening calculations.*

Panel A. Exposure scenarios

Scenario	IRS (mg day^{-1})	BW (kg)	EF (days year^{-1})	ED (years)	ET (h day^{-1})	SA (cm^2)	AF (mg cm^{-2})	ATnc (days)	ATnc (hours)
Child resident/public access	200	15	365	6	24	2800	0.2	2190	52560
Adult resident/public access	50	70	365	30	24	5700	0.2	10950	262800
Adult outdoor/maintenance worker	50	70	250	25	8	3300	0.2	9125	219000

IRS, soil ingestion rate; BW, body weight; EF, exposure frequency; ED, exposure duration; ET, exposure time; SA, exposed skin area; AF, soil adherence factor; ATnc, non-cancer averaging time. Cancer averaging time was 25,550 days or 613,200 h for every receptor. The worker scenario was evaluated only for the 18 urban sites.

Panel B. Toxicity values and endpoint treatment

Element	Analyte or assumption	RfDo (mg kg^{-1} day^{-1})	RfCi (mg m^{-3})	SFo (per mg kg^{-1} day^{-1})	IUR (per µg m^{-3})	ABSd	Use in screening model
As	Arsenic inorganic	3.00×10^{-4}	1.50×10^{-5}	1.5	0.0043	0.03	HQ: oral, dermal, inhalation; CR: oral, dermal, inhalation. Total As treated conservatively as inorganic.
Be	Beryllium and compounds	0.002	2.00×10^{-5}	—	0.0024	—	HQ: oral, inhalation; CR: inhalation.
Cd	Cadmium diet	1.00×10^{-4}	1.00×10^{-5}	—	0.0018	0.001	HQ: oral, dermal, inhalation; CR: inhalation.
Co	Cobalt and compounds	3.00×10^{-4}	6.00×10^{-6}	—	0.009	—	HQ: oral, inhalation; CR: inhalation.
Cr	Total chromium	—	—	—	—	—	Excluded from Cr-specific HQ/HI/CR because oxidation-state speciation was unavailable.
Cu	Copper	0.04	—	—	—	—	HQ: oral only.
Fe	Iron	—	—	—	—	—	No route-specific endpoint in the retained screening model.
Mn	Manganese non-diet	0.024	5.00×10^{-5}	—	—	—	HQ: oral and inhalation.
Ni	Nickel soluble salts	0.02	1.00×10^{-5}	—	2.60×10^{-4}	—	HQ: oral, inhalation; CR: inhalation.
Pb	Lead and compounds	—	—	—	—	—	Excluded from generic HQ/CR; evaluated as a separate concentration screening ratio.
V	Vanadium and compounds	0.00504	1.00×10^{-4}	—	—	—	HQ: oral and inhalation; no cancer endpoint assigned to total V.
Zn	Zinc and compounds	0.3	—	—	—	—	HQ: oral only.

RfDo, oral reference dose; RfCi, inhalation reference concentration; SFo, oral slope factor; IUR, inhalation unit risk; ABSd, dermal absorption fraction; HQ, hazard quotient; HI, hazard index; CR, cancer risk. The oral relative bioavailability factor was 1.0 for every element. Blank entries indicate that no value was applied for that route or endpoint.

Panel C. Common screening constants

Parameter	Value	Unit	Application
PM_{10} concentration	19.5025	μg m^{-3}	Inhalation exposure calculation
Respirable soil fraction	0.5	fraction	Inhalation exposure calculation
Pb resident/public-access comparator	200	mg kg^{-1}	Resident/public-access Pb ratio denominator
Pb worker comparator	800	mg kg^{-1}	Adult-worker Pb ratio denominator
HI classification threshold	1	dimensionless	HI classification
CR lower band breakpoint	1.00×10^{-6}	probability	Cancer-risk band classification
CR upper band breakpoint	1.00×10^{-4}	probability	Cancer-risk band classification

Table S6. Adult-worker screening summary and stability of the principal HI and CR drivers.

Panel A. Full-design (R4) adult outdoor/maintenance-worker screening summary

Receptor	*n*	Median HI [P05–P95]	Sites with HI ≥ 1, *n* (%)	Median CR [P05–P95]	Median Pb ratio [P05–P95]	Sites with Pb ratio ≥ 1, *n* (%)
Adult outdoor/maintenance worker	18	0.101 [0.078–0.138]	0 (0.0%)	5.44×10^{-6} [3.99×10^{-6}–8.98×10^{-6}]	0.130 [0.050–0.376]	0 (0.0%)

The worker scenario was evaluated for the 18 urban sites. Pb ratios used the worker comparator of 800 mg kg^{-1}. HI and CR are screening quantities and are not site-specific risk estimates.

Panel B. Probability of retaining the principal R4 element driver

Receptor	Design	Eligible sites, *n*	Mean probability of retaining R4 principal HI driver	Mean probability of retaining R4 principal CR driver
Child resident/public access	R1	26	99.0%	100.0%
	R2	26	100.0%	100.0%
	R3	26	100.0%	100.0%
Adult resident/public access	R1	26	92.3%	100.0%
	R2	26	94.2%	100.0%
	R3	26	100.0%	100.0%
Adult outdoor/maintenance worker	R1	18	94.4%	100.0%
	R2	18	94.4%	100.0%
	R3	18	100.0%	100.0%

No HI classification switch or cancer-risk-band switch occurred for any eligible receptor–site combination under R1–R3. No Pb classification switch occurred for the worker scenario. Resident Pb classification-switch probabilities are reported in Table 4 and Figure 5 of the main article.
